# Plant–microbe synergy: employing coastal plant bacteria for wheat prosperity under combined saline and heat stress

**DOI:** 10.1007/s00253-025-13678-w

**Published:** 2025-12-24

**Authors:** Ivana Staiano, Stefany Castaldi, Ermenegilda Vitale, Carmen Arena, Rachele Isticato

**Affiliations:** 1 Department of Biology, University of Naples Federico II, Complesso Universitario Monte S. Angelo, Naples, Italy; 2 National Biodiversity Future Center (NBFC), Palermo, 90133 Italy; 3 Interuniversity Center for Studies On Bioinspired Agro-Environmental Technology (BAT Center), Portici, NA Italy

**Keywords:** Plant growth-promoting rhizobacteria, Multi-stress environment, Abiotic stress, Wheat growth promotion, Eco-friendly, Microbial consortia

## Abstract

**Abstract:**

Environmental stresses due to climate changes, such as high temperatures and land degradation, significantly impact crop yield, making innovative strategies necessary to increase plant stress tolerance. This study investigates the potential of plant growth-promoting rhizobacteria (PGPR) to enhance wheat resilience under multiple environmental stresses, such as high salinity and temperature. For this, 15 bacterial strains were isolated from the rhizosphere and roots of *Pancratium maritimum* and screened for their ability to withstand high salinity (50–600 mM NaCl) and elevated temperatures (up to 42 °C). The isolates were identified by 16S rRNA sequencing and tested for their PGP traits under combined abiotic stresses. Most of the strains exhibited PGP features, such as biofilm formation, phosphate solubilization, and phytohormone production. To enhance the growth of wheat plants, used as a model crop of commercial interest, three different consortia were designed and tested in vitro. The consortium (CONSIII), composed of *Serratia marcescens* ERA6, *Enterobacter cloacae* ERA9, and *Bacillus proteolyticus* ESOB2, provided synergistic effects that led to an enhancement in plant growth and stress resilience in vitro. This positive effect was confirmed in pot trials under double abiotic stress (37 °C, 132 mM NaCl), where CONSIII was able to boost the root and shoot growth, increase chlorophyll and carotenoid content, and enhance antioxidant activity, mitigating reactive oxygen species accumulation. These findings underscore the potential of PGPR consortia as bioinoculants for sustainable agriculture, demonstrating their effectiveness in the simultaneous presence of salinity and heat stresses—a challenging and under-investigated environmental scenario.

**Key points:**

• *PGPR strains isolated from Pancratium maritimum rhizosphere are able to grow and exhibit PGP traits under combined salinity and heat conditions*

• *The formulated consortium of PGPR strains (CONSIII) significantly enhances wheat growth and stress resilience under a multi-stress environment*

• *CONSIII increases plant biomass, pigment content, and antioxidant activity, proving its value as a sustainable bioinoculant*

**Supplementary information:**

The online version contains supplementary material available at 10.1007/s00253-025-13678-w.

## Introduction

Over the last decades, the cumulative impact of human activity on the planet has led to numerous extreme environmental conditions being introduced into ecosystems and agricultural lands (Grimm et al. 2008; Teuling 2018). These conditions encompass climate change-driven extreme and fluctuating weather events, such as heat waves and prolonged droughts, in combination with adverse soil conditions (saline, alkaline, and/or acidic soils) (Rosenzweig and Hillel 2000), anthropogenic contaminants (heavy metals, microplastics, pesticides, antibiotics, and persistent organic pollutants) (Kallenborn et al. 2012), radiation (UV) (McKenzie et al. 2011), limited nutrient availability (Brouder and Volenec 2008), and elevated levels of airborne molecules and gases (ozone, combustion particles, CO_2_) (Chakraborty et al. 2000). Plants are continuously exposed to these multi-stress factors, which adversely affect their reproduction, survival, and resistance to phytopathogens, ultimately contributing to ecosystem deterioration and a reduction in crop yields (Hamann et al. 2021; Lee et al. 2023). Since the frequency and intensity of these abiotic stress combinations are expected to rise in the coming years (Alizadeh et al. 2020; Zandalinas et al. 2022), there is a strong need to understand their combined effects on plants. While previous studies have traditionally focused on the impact of individual stressors, the scientific community is increasingly shifting toward studying the complexity of multiple stressors affecting plants in natural environments and on the search for eco-friendly strategies to address the problem (Coolen et al. 2016; Defo et al. 2019). In this context, there is an increasing interest in endophytic microbial communities as microbial-based strategies for enhancing crop yield in multi-stress conditions. A specific group of microorganisms known as plant growth-promoting rhizobacteria (PGPR) have garnered attention for their beneficial effects on plant growth. PGPR positively influences plant growth and offers promising and sustainable solutions to increase plant biomass production under multi-stress environments (Lindemann et al. 2016; Umesha et al. 2018). These microorganisms directly contribute to plant growth by synthesizing phytohormones such as indole-3-acetic acid (IAA), gibberellins, and cytokinins and performing functions such as phosphate solubilization and nitrogen fixation. In addition, these beneficial bacteria release various secondary metabolites and volatile compounds that inhibit phytopathogen growth and alleviate abiotic stresses such as high temperature and salinity (Semwal et al. 2025). High salinity and elevated temperatures can severely impair plant performance and crop productivity. Saline soils reduce seed germination and inhibit root and shoot elongation. They also alter biomass partitioning and accelerate leaf senescence, ultimately leading to decreased growth and yield (Shilev 2020; Zheng et al. 2022). Similarly, high temperatures alter evapotranspiration and water balance, resulting in oxidative stress, metabolic dysfunction, and reduced photosynthesis (Chaves et al. 2003; Suzuki et al. 2016). Plants employ multiple adaptive strategies, such as altering molecular mechanisms involving proteins, antioxidants, metabolites, regulatory factors, and membrane lipids (Kai and Iba 2014). However, these mechanisms often prove insufficient in the face of the increasing frequency of combined heat and salinity stress associated with climate change (Naylor and Coleman-Derr 2018). In this context, we focused on the isolation and characterization of halo- and thermotolerant PGPR since, under ongoing climate change, agricultural soils are increasingly exposed to both high temperatures and salt accumulation, making their combined effect one of the most pressing challenges for sustainable crop production (Naylor and Coleman-Derr 2018). In line with this premise, we analysed the microbiome associated with the sea daffodil *Pancratium maritimum* L., a perennial species from the *Amaryllidaceae* family that grows in the Mediterranean coast’s nutrient-deficient, saline, sandy soils. This species is adapted to withstand extreme environmental stresses like high temperatures, severe sunlight, and extremely low availability of freshwater (Defo et al. 2019). The fact that plants can cope with such harsh conditions suggests that it is, at least in part, mediated through interactions with halotolerant PGPRs, making it a prime candidate for isolating stress-tolerant bacterial strains. To this aim, 15 PGPR strains were isolated from the root-associated microbiome, selecting those capable of withstanding high temperatures and salinity. Their plant growth-promoting (PGP) activities were assessed, and five top-performing strains were further evaluated in vitro for their ability to enhance plant growth under temperature and salinity stress using wheat (*Triticum durum* cv. Creso) as a model crop.

## Material and methods

### Sampling and isolation of bacteria from *P. maritimum* roots

*P. maritimum* L. roots were collected from a beach in Diamante (Cosenza, Italy) in sterile containers and stored in sterile 1X PBS (phosphate saline buffer) at 4 °C until processed. According to the International Union for Conservation of Nature (IUCN, Cambridge, UK), this plant is not endangered, and the sampling method was non-destructive. Exophytic bacteria were isolated from the 1X PBS storage solution containing root material. To obtain endophytic bacteria, roots were washed in 70%v/v ethanol for 5 min, followed by several rinses with sterile water. The final rinse (0.1 mL) was plated on Luria–Bertani (LB: 8 g L⁻^1^ NaCl, 10 g L⁻^1^ tryptone, 5 g L⁻^1^ yeast extract) agar to confirm surface disinfection. Surface-disinfected roots (1.0 g) were homogenized in 10 mL sterile 1X PBS. To isolate bacteria, serial dilutions (up to 10⁻^6^) of the homogenate roots and of the roots’ storage solution were plated onto LB agar (15 g L⁻^1^ agar) supplemented with three different NaCl concentrations (50 mM, 132 mM, and 330 mM). In particular, the concentration of 132 mM NaCl corresponds to an electrical conductivity of approximately 12–13 dS m⁻^1^ in solution, a level that lies within the range typically used to define highly to strongly saline conditions in soils (USDA NRCS 2014). This salinity level is sufficiently high to impair germination and plant growth, yet not so extreme as to cause immediate plant mortality, making it suitable for evaluating the mitigating effects of PGPR under realistic and biologically meaningful salt-stress conditions (Mosallanejad et al. 2025). The plates were incubated at 25 °C, 37 °C, and 42 °C for 24–48 h. Colonies displaying distinct morphologies were selected, purified by streaking on fresh LB agar, and characterized by colony colour, shape, size, margin, and appearance. Every isolate was also cultivated on DSM agar plates (8 g L^−1^ nutrient broth, 1 g L^−1^ KCl, 1 mM MgSO_4_, 1 mM Ca(NO_3_)_2_, 10 μM MnCl_2_, 1 μM FeSO_4_, Sigma-Aldrich, Darmstadt, Germany) (Vittoria et al. 2023a) at the three temperatures to assess spore-forming ability (Nicholson and Setlow 1990). Glycerol stocks (20%v/v) of all isolates were stored at − 80 °C.

### 16S rRNA sequencing and phylogenetic analysis

Chromosomal DNA was extracted using the DNeasy PowerSoil kit (Qiagen, Hilden, Germany) following the manufacturer’s protocol. The 16S ribosomal RNA (rRNA) gene (~ 1500 base pairs) was amplified by polymerase chain reaction (PCR) using chromosomal DNA as a template and the primers 8 F (5′–AGTTTGATCCTGGCTCAG-3′, annealing at positions + 8 to + 28) and 1517R (5′-ACGGCTACCTTGTTACGACT-3′, annealing at positions + 1497 to + 1517). PCR reactions were performed as described by Haiyambo et al. (2015) using an Esco Swift MaxPro Thermal Cycler (Esco Lifesciences, Singapore). Amplicons were purified with the QIAquick PCR Purification Kit (Qiagen, Hilden, Germany) according to the manufacturer’s protocol and sequenced at the BMR Genomics sequencing facility (Padova, Italy). Sequence quality was verified with SeqTrace (minimum Phred score 30, minimum 20 consecutive bases) (Stucky 2012). Trimmed paired sequences were analysed via BLASTn (available at NCBI, Bethesda, MD, USA https://blast.ncbi.nlm.nih.gov/Blast.cgi). The phylogenetic tree (Fig. S1, Supplementary Materials) was constructed using the neighbour-joining method (total branch length = 9.418, bootstrap = 1000 replicates). Evolutionary distances were calculated with the Kimura two-parameter model, rate variation among sites was modelled with a gamma distribution (shape = 1.00), and pairwise deletion was applied. The final dataset comprised 1600 positions. Analyses were conducted in MEGA12 (Kumar et al. 2024) software (Arizona State University, Tempe, AZ, USA) using up to seven parallel computing threads. The 16S rRNA sequence of *Aquifex aeolicus* (AJ309733.1) served as the outgroup. All 16S rRNA sequences were deposited in the NCBI Sequence Read Archive and are identified by accession numbers listed in Table 1. All isolated and identified strains are maintained in the Micralab bacterial collection, Department of Biology, University of Naples Federico II, under the supervision of Prof. Rachele Isticato. Strains are stored as cryopreserved cultures at −80 °C in 20% v/v glycerol and are publicly accessible upon request to Rachele Isticato (isticato@unina.it).

**Table 1:** Taxonomical identification of the isolated strains throughout 16S rRNA blast analysis

Strain ID	Accession number	Nearest neighbour from NCBI with accession number	Similarity (%)
ERA1	PV239528	*Enterobacter hormaechei* subsp. *xiangfangensis* (NR 126208.1)	99.67
ERA2	PV239529	*Pseudomonas juntendi* (NR 180457.1)	99.77
ERA3	PV239530	*Bacillus paramycoides* (NR 157734.1)	98.71
ERA4	PV239531	*Kosakonia cowanii* (NR 025566.1)	98.53
ERA5	PV239532	*Pseudomonas taiwanensis* (NR 116172.1)	99.70
ERA6	PV239533	*Serratia marcescens* (NR 114043.1)	99.40
ERA7	PV239534	*Pseudomonas plecoglossicida* (NR 114226.1)	99.55
ERA9	PV239535	*Enterobacter cloacae* subsp. *dissolvens* (NR 118011.1)	98.50
ESOA	PV239540	*Enterobacter cloacae* subsp. *dissolvens* (NR 044978.1)	98.97
ESOB1	PV239541	*Enterobacter cloacae* (NR 113615.1)	99.03
ESOB2	PV239542	*Bacillus proteolyticus* (NR 157735.1)	99.70
ERAS1	PV239536	*Bacillus cabrialesii* (NR 180419.1)	99.20
ERAS2	PV239537	*Bacillus safensis* (NR 113945.1)	99.34
ERAS3	PV239538	*Bacillus stercoris* (NR 181952.1)	99.42
ERAS4	PV239539	*Rossellomorea aquimaris* (NR 025241.1)	99.04

### In vitro assessment of plant growth promoting traits

#### Evaluation of the physiological properties

Swarming motility was tested using LB plates with 0.7%w/v of agar by measuring the radius of the grown colony spot-inoculated with 5 µL of fresh bacterial culture, after incubation overnight at the three temperatures. Biofilm production was tested using the Congo Red Agar (CRA) method as reported in Lee et al. (2016). Five microlitres of fresh bacterial culture was spot inoculated on CRA plates and incubated at the respective temperature. A positive result for biofilm formation was indicated by black colonies with a dry crystalline consistency. Conversely, smooth orange to red colonies and colonies that remained pink indicated non-biofilm producers.

#### Phosphate solubilization

A modified Pikovskaya (PVK) medium (Schoebitz et al. 2013) was used to assess the phosphate-solubilizing capacity of bacterial isolates through the dissolution of calcium phosphate (Ca_3_(PO_4_)_2_). Five microlitres of exponential-phase bacterial cultures was applied onto PVK agar containing three NaCl concentrations (50 mM, 132 mM, and 330 mM) and incubated for 10 days at 25 °C, 37 °C, and 42 °C. The formation of a transparent halo zone surrounding the bacterial colonies indicated phosphate solubilization.

#### Indole-3-acetic acid (IAA) production

Indole-3-acetic acid (IAA) production was quantified using a modified method based on Gordon and Weber (1951). Bacteria were grown in LB broth with or without 0.5 mg mL^−1^ tryptophan (Sigma-Aldrich, Darmstadt, Germany) and supplemented with three NaCl concentrations (50 mM, 132 mM, and 330 mM) for 48 h at 25 °C, 37 °C, and 42 °C with shaking at 150 rpm. After centrifugation (7,000 rpm, 4 °C, 10 min), 67 µL supernatant were mixed with 133 µL Salkowski reagent (H_2_O:H_2_SO_4_:FeCl_3_ 0.5 M in a 50:30:1 ratio) and incubated for 30 min at room temperature. Absorbance at 530 nm was measured using a Synergy HTX Multi-Mode Microplate Reader (BioTek, Winooski, VT, USA). Uninoculated medium with Salkowski reagent served as a negative control. IAA production was quantified using a standard curve generated from serial dilutions of IAA (Sigma-Aldrich, Darmstadt, Germany) (from 500 to 3.9 µg µL^−1^, eight stocks obtained with serial dilutions and tested in triplicate) (Gordon and Weber 1951; Tsavkelova et al. 2006).

#### Detection of ammonia

Isolates were cultured in 1% w/v peptone broth supplemented with NaCl at concentrations of 50 mM, 132 mM, and 330 mM for 72 h at 25 °C, 37 °C, and 42 °C with shaking at 150 rpm. Cultures were centrifuged (7000 g, 4 °C, 10 min), and 20 µL supernatant was added to 176 µL water + 4 µL Nessler’s reagent in a 96-well plate following a modified Demutskaya and Kalinichenko (2010) protocol. Ammonia production was quantified by measuring optical density at 450 nm using a Synergy HTX Multi-Mode Microplate Reader (BioTek, Winooski, VT, USA). The concentration of ammonia was estimated based on a standard curve of ammonium sulphate prepared with nine concentrations ranging from 3.9 to 1000 µg L^−1^, obtained with serial dilutions of the initial ammonium sulphate stock and tested in triplicate (Demutskaya and Kalinichenko 2010).

#### DPPH assay

The α,α-diphenyl-β-picrylhydrazyl (DPPH) free radical scavenging method was used to evaluate the potential production of biomolecules with antioxidant activity of the isolated strains as described by Xiang et al. (2018). Briefly, 0.2 mL of fresh bacterial culture, grown at the different conditions of salinity and temperature (50 mM, 132 mM, and 330 mM, and 25 °C, 37 °C, 42 °C), were incubated in a final volume of 1 mL of methanol containing 0.1 mM of freshly prepared DPPH (dissolved in methanol). The reaction was allowed to proceed for 30 min in the dark at room temperature. The DPPH free radical scavenging activity was then monitored by determining the absorbance at 517 nm and calculated according to the following equation:

$$\text{DPPH radical scavenging activity }\left(\mathrm{\%}\right)=\left(1- \frac{{\mathrm{Abs}}_{\mathrm{sample}}}{{\mathrm{Abs}}_{\mathrm{control}}}\right)\bullet 100$$

where Abs_sample_ is the absorbance of the reacted mixture of DPPH with the extract sample and Abs_control_ is the absorbance of the DPPH solution (Vittoria et al. 2023b).

### Screening for hydrolytic enzymatic activity

The hydrolytic enzyme assays (amylase, protease, and cellulase) were performed on solid media. All enzymatic activities were performed by growing the bacterial isolates in LB broth for 24 h at 37 °C. After incubation, 5 µL of each fresh bacterial culture was spot inoculated on the different assay plates complemented with different salt concentrations (50 mM, 132 mM, and 330 mM) and incubated at the three temperatures (25 °C, 37 °C, 42 °C). To detect the amylase activity, starch agar plates were used as previously reported (Nimisha et al. 2019). After 72 h of incubation, the plates were flooded with Gram’s iodine solution, and the hydrolysis of starch was observed as a colourless zone around grown colonies. For the proteolytic activity, isolated bacteria were spot inoculated on skimmed milk agar (SMA) plates. The formation of clear halos around the colony was confirmed as proteolytic activity (Morris et al. 2012). For the detection of cellulase and xylanase activities, xylanase production medium (XPM) (Meddeb-Mouelhi et al. 2014) agar plates were used with 0.5%w/v xylan from beechwood (Megazyme, Bray, Ireland) and a minimal medium with 0.5%w/v carboxymethylcellulose (CMC) (Hankin and Anagnostakis 1977) as sole carbon sources. After incubation for 3 days, hydrolysis zones were visualized by flooding the plates with 0.1%w/v Congo Red aqueous solution for 30 min and then destained by washing twice with 1 M NaCl. Plates without carbon source were used as non-substrate controls. Transparent-yellowish hydrolytic zones around the colonies were considered positive.

### Dual-culture method for the evaluation of antifungal activity

The isolated strains were examined in vitro for antifungal activity against pathogenic fungus *Parastagonospora nodorum* (Ragucci et al. 2023) and *Pyrenophora tritici-repentis* (Carmona et al. 2006). The two fungal strains used in this study were kindly supplied by Prof. Marcelo Anibal Carmona (Facultad de Agronomía, Cátedra de Fitopatología, Universidad de Buenos Aires, Buenos Aires, Argentina) (Hafez et al. 2023). Pure cultures were grown for 5 days at 25 ± 1 °C on PDA (potato dextrose—BD DIFCO™ Becton, Dickinson and Company, Franklin Lakes, NJ, USA—18 g L^−1^ agar) and deposited in the fungal culture collection of the Biology Department of the University of Naples Federico II, Italy. The in vitro antifungal bioassays were carried out based on the dual-culture method as previously described by Khamna et al. (2009) with some modifications. Fungal plugs, 5 mm in diameter, were placed at the centre of PDA plates. On the opposite side, 5 µL of bacterial strains grown overnight in LB broth were added 1.5 cm from the fungal disc. Control plates contained only fungal plugs without bacterial inoculation. All plates were incubated at 28 °C for 5 days, and the experiment was performed in triplicate. Inhibition of pathogenic fungal growth by bacterial strains was assessed using a stereoscopic microscope at 10X magnification (Castaldi et al. 2023).

### Compatibility assay

Pairwise compatibility among isolates was evaluated by a modified agar diffusion test. Each strain was cultured in 5 mL LB overnight at 37 °C (150 rpm), then diluted into soft LB agar (0.7% w/v) to ~ 10^8^ cells mL^−1^, solidified, and then 10 µL of another strain’s overnight culture was spotted on top. Plates were incubated at 37 °C and monitored daily for 7 days. Compatibility was defined by colony overlap, while incompatibility was defined by a clear inhibition zone around the colony. Each combination was replicated three times using the same bacteria as a control for compatibility.

### Plant material and in vitro growth conditions

All experiments were carried out with wheat seeds (*T. durum *cv. Creso) that were kindly provided by Prof. Sheridan Lois Woo (Department of Pharmacy, University of Naples Federico II) (Silletti et al. 2021). Seed sterilization followed the protocol described by Barbulova et al. (2005). Unsynchronized seedlings were removed 5 days after sowing on H_2_O agar Petri dishes under sterile conditions. Synchronized germinated seedlings were incubated with bacterial suspension if necessary and transferred to Petri dishes containing H_2_O agar (1%w/v) supplemented with different NaCl concentrations. The plates were incubated in the dark at 25 °C or 37 °C with different salt concentrations for 7 days.

### Effects of PGPR on the growth of wheat

Bacteria were grown overnight in LB broth at 37 °C at 150 rpm; the pellets were washed three times with sterile 1X PBS and then resuspended in a final volume of 10 mL of sterile 1X PBS to be quantified with a Bürker chamber (Sigma-Aldrich, Darmstadt, Germany, BR719505) under an optical microscope (Olympus BH-2 with 100 × lens, Tokyo, Japan) and diluted to 10^8^ cells mL^−1^ in 30 mL of 1X PBS for seed-biopriming. Synchronized seedlings were incubated with bacterial suspensions overnight at room temperature with continuous shaking and then transferred to Petri dishes with H_2_O agar (1%w/v) supplemented with different concentrations of NaCl (0 mM or 132 mM) and incubated at 25 °C or 37 °C in the darkness.

### Pot experiments

Sterilized seeds (as above) were inoculated with bacteria (10^8^ cells mL^−1^ in 30 mL of 1X PBS) when needed while controls were incubated in sterile 1X PBS; a schematic representation of the experiment is described in Supplemental Table S1. After the overnight incubations, ten seeds were sown in each pot at a 1 cm depth in double autoclaved horticulture soil (Compo Sana® Universal Potting Soil, Compo GmbH, Münster, Germany) and irrigated regularly to prevent water stress. Plants were cultivated for 30 days in a controlled growth chamber with a light intensity of 200 μmol m^−2^ s^–1^ at 25 °C or 37 °C with a 16 h:8 h, light/night cycle.

### Evaluation of biochemical parameters in plants

#### Total chlorophyll and carotenoid content in leaves

Five leaves per treatment were collected to determine total chlorophyll (*a* + *b*) and total carotenoid (*x* + *c*) concentrations following the procedure reported by Petrillo et al. (2022). Fresh samples (10 mg) were powdered in liquid nitrogen and treated with ice-cold 100% acetone and centrifuged (Labofuge GL, Heraeus Sepatech, Hanau, Germany) at 5000 rpm for 5 min. The absorbance of supernatants was read using a spectrophotometer (Cary 100 UV–VIS, Agilent Technologies, Santa Clara, CA, USA) at 470, 645, and 662 nm. The pigment concentration was determined through the Lichtenthaler equations and expressed in mg g^−1^ of fresh weight (mg g^−1^ FW) (Lichtenthaler 1987).

#### Antioxidant activity in leaves and roots

The antioxidant activity was determined in leaves and roots on six replicates per treatment performing the DPPH free radical scavenging activity assay, according to Castaldi et al. (2024). Briefly, fresh samples (50 mg) were powdered in liquid nitrogen and extracted in methanol overnight. Then, the extracts were centrifuged at 14,000 rpm for 15 min at 4 °C, mixed with a 6 × 10^−5^ M DPPH methanolic solution, and incubated at 37 °C for 20 min. The absorbance was measured at 515 nm with a spectrophotometer (BioTek Synergy HTX Multimode Reader, Agilent Technologies, Winooski, VT, USA) and converted in percentage of DPPH radical inhibition through the formula:

$$\text{DPPH radical scavenging activity }\left(\mathrm{\%}\right)=\left(1- \frac{{\mathrm{Abs}}_{\mathrm{sample}}}{{\mathrm{Abs}}_{\mathrm{control}}}\right)\bullet 100$$

where *A*_control_ is blank absorbance on the DPPH methanolic solution and *A*_sample_ is the sample absorbance.

### Statistical analysis

All statistical analyses were performed using GraphPad Prism (GraphPad Prism 8.0.1 Software, San Diego, CA, USA), and the data were expressed as the mean ± SEM (standard error of the mean). As indicated in figure legends, differences among groups were compared with one-way ANOVA test by Tukey’s test (*α* = 0.05) or with unpaired *t-*test (two-tailed *α* = 0.05). Statistical significance was defined as *p* < 0.05.

## Results

Root samples of *P. maritimum* L. were collected to isolate bacteria capable of withstanding multiple stress conditions. Exophytic bacteria were isolated by washing the roots in 1X PBS, while endophytic bacteria were obtained following surface sterilization and subsequent root grinding. The isolated bacteria were subjected to a gradual enrichment process involving incubation under increasing temperatures and salinity levels. Specifically, the temperatures tested included 25 °C as a control and 37 °C and 42 °C, which represent extreme summer temperatures commonly observed in the Mediterranean region (Tejedor et al. 2024). Salinity concentrations tested were 50, 132, 330, and 600 mM of NaCl, corresponding to low-salinity conditions, typical slightly saline environments, moderately saline environments, and high salinity environments, respectively (van Zelm et al. 2020). Through this approach, 12 endophytic (ERA strains) and three exophytic (ESO strains) isolates were selected based on their unique cultural characteristics, including colony size, shape, elevation, surface texture, consistency, pigmentation, and growth response to varying salt concentrations and temperatures (Supplemental Table S2). As shown in Supplemental Table S2, 10 of the 15 strains of bacteria were tolerant to up to 600 mM NaCl, while 9 strains grew at temperatures of up to 42 °C. Notably, strains ERA3, ESOB2, ERAS1, ERAS3, and ERAS4 were identified as spore-forming bacteria. Amplification and sequencing of the 16S rRNA gene were performed to achieve taxonomic identification of all isolated bacteria. BLAST analysis of the sequences against the NCBI database (Table 1) found strains mainly belonging to three genera: *Bacillus*, *Pseudomonas*, and *Enterobacter* (Table 1).

Analysis of the phylogenetic tree, constructed using the neighbour-joining (NJ) algorithm, also clustered the strains into three, representing *Bacillus*, *Pseudomonas*, and *Enterobacter*, as shown in Supplemental Fig. S1.

### Screening for hydrolytic enzyme activity

**Fig. 1:**
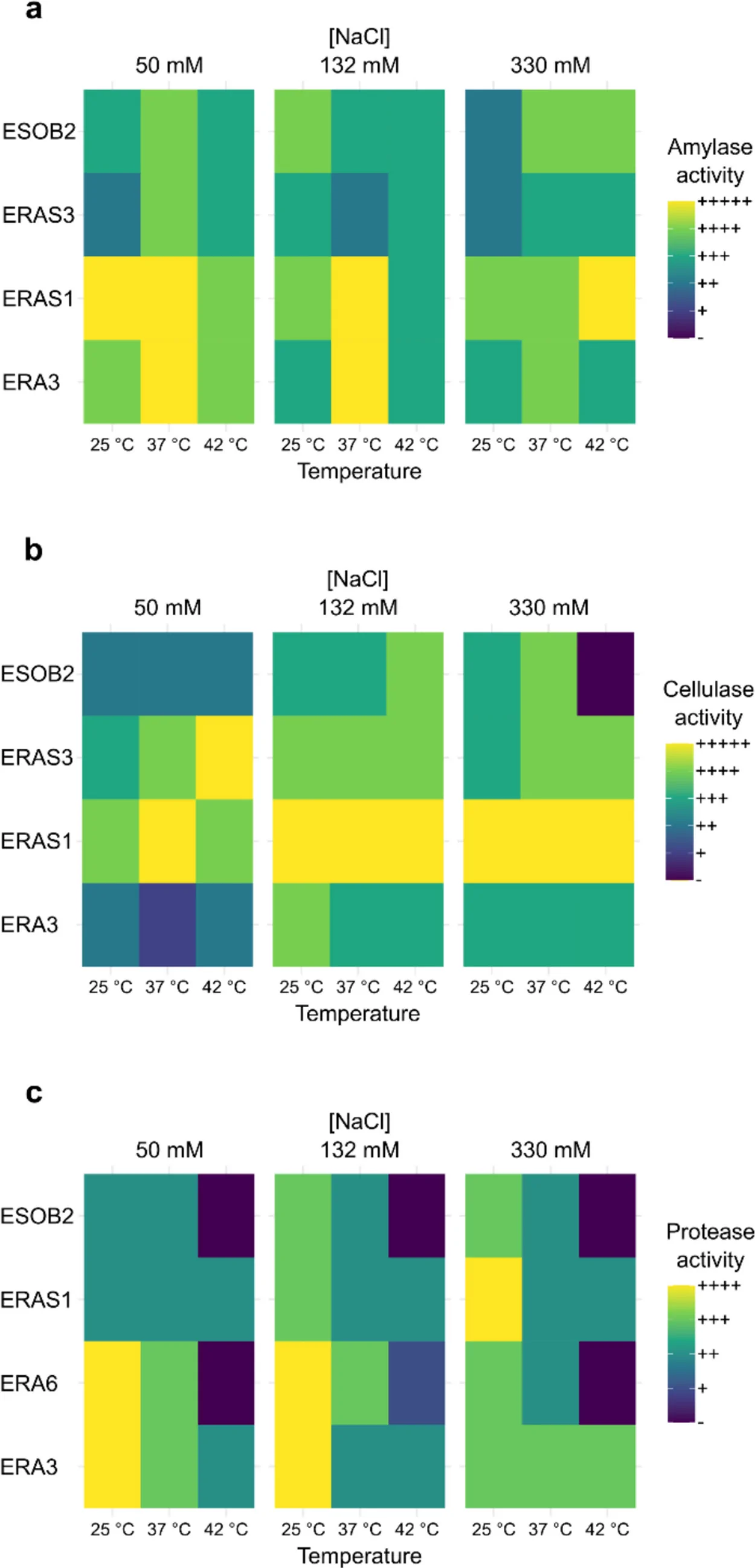
Higher hydrolytic activity observed among 15 isolated strains (only the 4 most efficient strains are reported) under temperature and salt stress. Results of amylase production, (**a**) cellulase production, (**b**) and protease production (**c**) are reported as means of at least three replicates (*p* < 0.05) and are expressed as no activity (–), hydrolysis zone diameter (hzd) < 5 mm (+), hzd ≥ 5 mm (+ +), hzd ≥ 10 mm (+ + +), hzd ≥ 15 mm (+ + + +), hzd ≥ 20 mm (+++++)

Beneficial rhizobacteria can produce and secrete hydrolytic enzymes in the soil, which can break down complex organic macromolecules, increasing the availability of essential nutrients for plant and soil bacterial communities. Given the importance of these enzymes as plant growth-promoting (PGP) factors, the ability of the 15 isolated strains to secrete hydrolytic enzymes was therefore evaluated. Figure 1 shows the positive results obtained from five bacterial strains (*Bacillus proteolyticus* ESOB2, *Bacillus stercoris* ERAS3, *Bacillus cabrialesii* ERAS1, *Bacillus paramycoides* ERA3, and *Serratia marcescens* ERA6) out of the 15 tested, on 3 representative enzymatic activities (protease, amylase, and cellulase) associated with PGPR. As illustrated in Fig. 1a, * B. cabrialesii* ERAS1 and *B. paramycoides* ERA3 exhibited the strongest amylase activity in all tested multi-stress conditions, underscoring their robust enzymatic potential. Meanwhile, *B. proteolyticus* ESOB2 and *B. stercoris* ERAS3 showed moderate to high activity levels. Cellulase activity, assessed through the degradation of carboxymethylcellulose (CMC), revealed similarly promising trends. As shown in Fig. 1b, * B. proteolyticus* ESOB2, *B. stercoris* ERAS3, and *B. paramycoides* ERA3 demonstrated medium-to-high cellulase activity under all conditions. Notably, *B. cabrialesii* ERAS1 stood out as the top-performing strain, exhibiting the highest cellulase activity under the combined temperature and salinity stress scenarios. Finally, the protease activity analysis, reported in Fig. 1c, revealed distinct trends. *S. marcescens* ERA6 and *B. paramycoides* ERA3 exhibited strong protease activity at 25 °C in the presence of 50 mM NaCl, although their activity declined under more extreme temperature and salinity conditions. Remarkably, *B. cabrialesii* ERAS1 was the only strain capable of maintaining a moderate activity level even under the harshest tested conditions (42 °C).

### Antagonistic activity of isolates against fungal plant pathogen

**Fig. 2:**
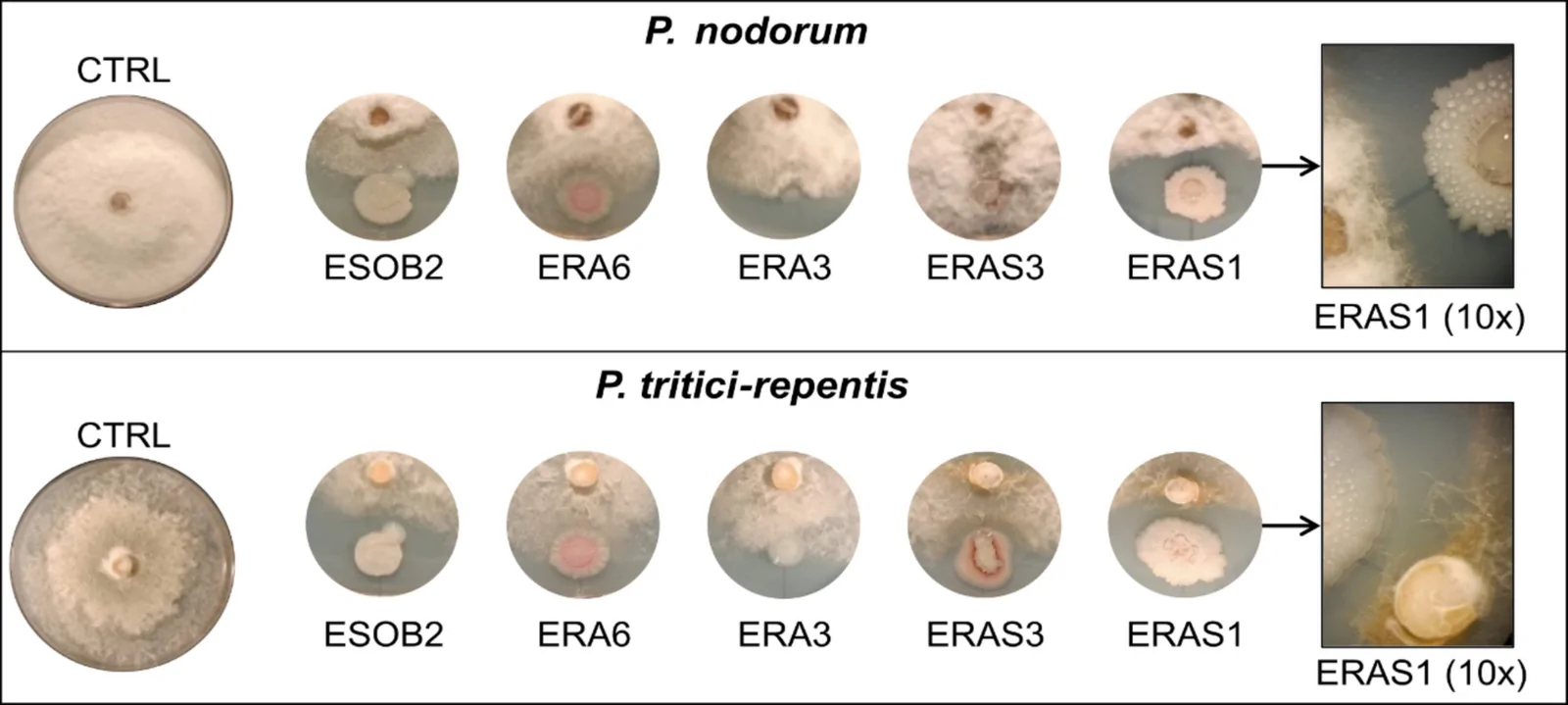
Antagonism assays on PDA medium. Photographs of dual-culture assay for in vitro inhibition of mycelial growth of *P. nodorum *and *P. tritici-repentis* by selected bacterial strains. PDA plates with only fungal inoculation (without bacteria) were used as controls (CTRL). Rectangles on the right represent the interaction zone of strain ERAS1 and the two fungi acquired with a stereoscopic microscope (10× magnification). All experiments were performed in triplicate with three independent trials

Given the promising hydrolytic activities of the strains *B. proteolyticus* ESOB2, *B. stercoris* ERAS3, *B. cabrialesii* ERAS1, *S. marcescens* ERA6, and *B. paramycoides* ERA3 (Fig. 1), their biocontrol activity was also evaluated. A dual culture assay was performed to evaluate their effectiveness against two wheat-pathogenic fungi, *P. nodorum* (Carmona et al. 2006) and *P. tritici-repentis* (Khamna et al. 2009) (Fig. 2). Both the phytopathogenic fungi utilized in our experiment infect wheat, the model plant used in this study, severely impairing photosynthesis and inducing necrosis in tissues, decreasing plant growth and yield. Furthermore, the high evolutionary potential of both fungi has allowed them to develop genetic resistance to environmental stresses and commonly used pesticides, making them significant threats in wheat cultivation worldwide (Downie et al. 2021; Guo et al. 2020).

**Fig. 3:**
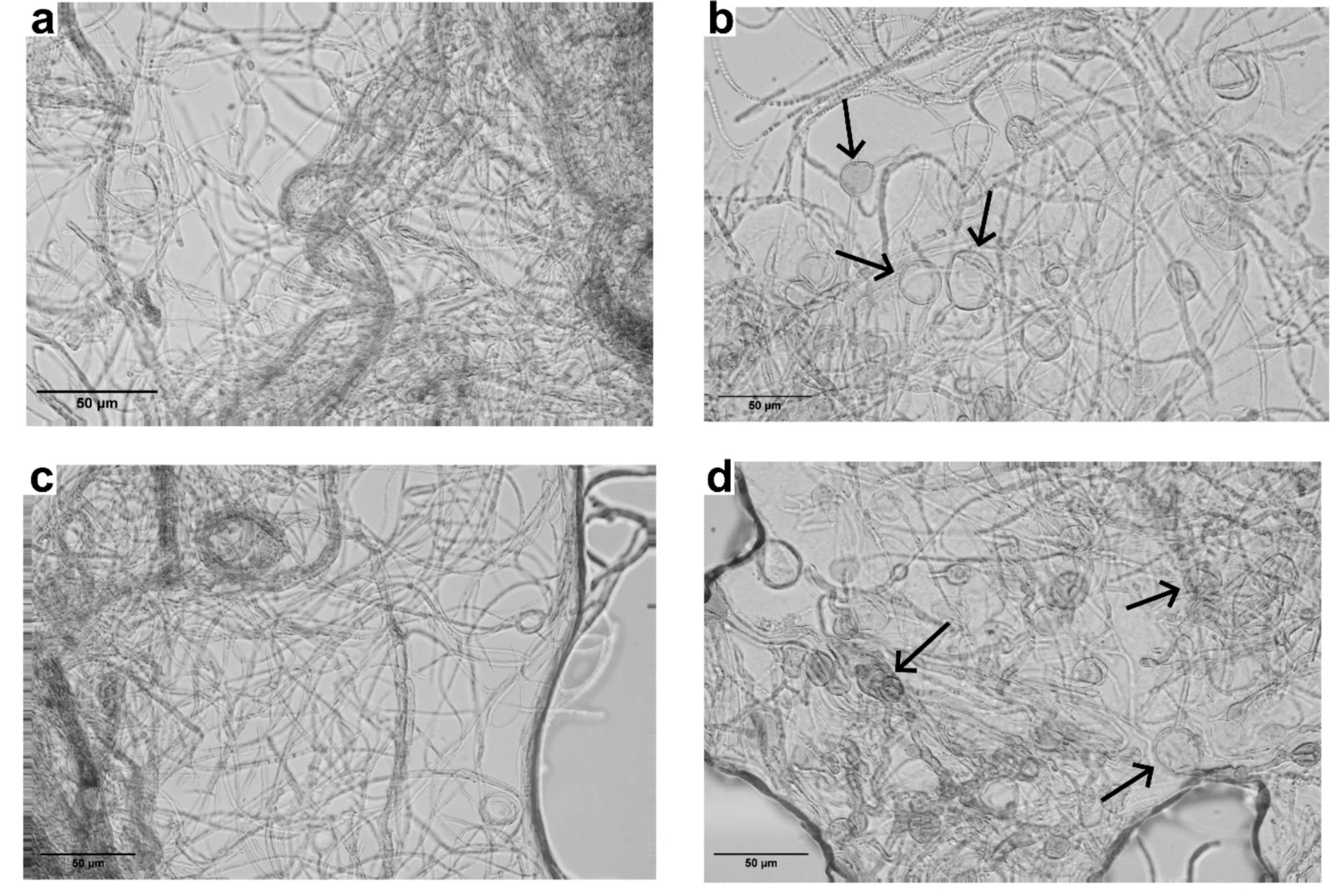
Observation of the effect of *B. cabrialesii* ERAS1 on the hyphae of *P. tritici-repentis*: **a** control, fungus grown without bacteria; **b** hyphae infected with the bacteria. Effect on *P. nodorum*; **c** control, fungus grown without bacteria; **d** hyphae infected with the bacteria. All the pictures are obtained with a phase contrast light microscope with 40× magnification. Arrows indicate the potential autophagosomes formed in the hyphae grown in the presence of the strain ERAS1

The dual-culture assays revealed variability in the inhibitory effects of the selected strains against the fungal pathogens, with some exhibiting strong antimicrobial activity while others showing limited or no effect. As illustrated in Fig. 2, strains *B. proteolyticus* ESOB2 and *B. stercoris* ERAS3 demonstrated slight inhibition against *P. nodorum* and showed more pronounced activity against *P. tritici-repentis*. In contrast, *B. paramycoides* ERA3 and *S. marcescens* ERA6 exhibited minimal activity against both phytopathogens. Interestingly, *B. cabrialesii* ERAS1, which showed the strongest hydrolytic activities under multi-stress conditions (Fig. 1), also exhibited notable antifungal activity, as evidenced by the inhibition zones observed between the bacterium and the fungal colonies (Fig. 2). To further determine the inhibitory action of *B. cabrialesii* ERAS1, a microscopic analysis of the co-cultured plates was performed to assess the morphology of the fungal hyphae (Fig. 3).

The analysis revealed a visible impact of the bacteria on the morphological characteristics of the hyphae. The panels in Fig. 3b and d (*P. tritici-repentis* and *P. nodorum* co-cultured with *B. cabrialesii* ERAS1, respectively) show that the bacteria caused alterations in comparison to the control (Fig. 3a and c, * P. tritici-repentis* and *P. nodorum* without *B. cabrialesii* ERAS1, respectively), while the hyphae of the treated sample exhibited a less uniform structure, with the presence of evident swellings in the cells that may indicate the occurrence of autophagosome-like structures, as previously described by Li et al. (2014).

### In vitro characterization of potential PGPR

**Fig. 4:**
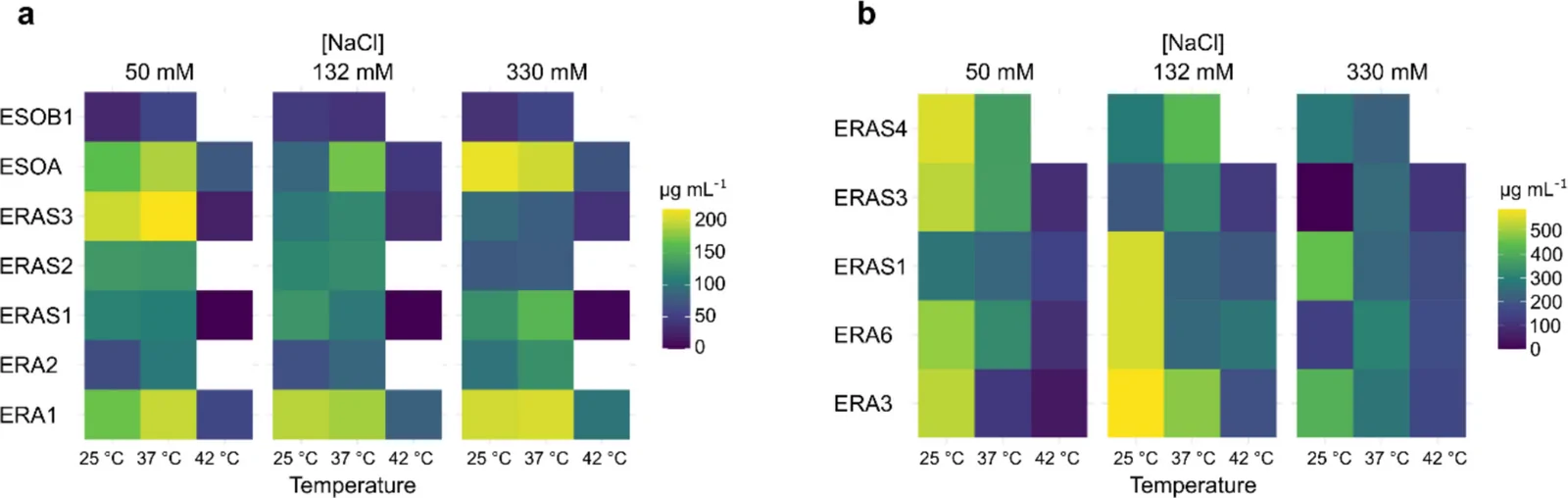
PGP traits evaluation under temperature and salt stress of top-performing strains. Results of IAA (**a**) and ammonia production (**b**) are reported as means of at least three replicates (*p* < 0.05). The presented data of IAA production are obtained by growing the bacteria in the presence of tryptophan (0.5 mg mL^−1^) as reported above. Blank spaces in the figure represent the strains that were not able to grow in the defined media at 42 °C

The 15 bacterial isolates were then assessed for their plant growth-promoting traits under the selected multi-stress conditions, including their abilities for swarming motility, biofilm production, phosphate solubilization, and polymer hydrolysis as previously described by Vasseur-Coronado et al. (2021). Among these, only the strains *Enterobacter cloacae* ESOA and* B. proteolyticus* ESOB2 demonstrated swarming motility, suggesting their potential for root colonization (Supplemental Table S2). Seven isolates (*Enterobacter hormaechei* ERA1, *B. paramycoides* ERA3, *Kosakonia cowanii* ERA4, *S. marcescens* ERA6, *E. cloacae* ERA9, *E. cloacae* ESOA, *Enterobacter cloacae* ESOB1) were capable of biofilm production, a key feature for adhesion to the root surface. Phosphate solubilization, an important trait for improving nutrient availability (Supplemental Table S3), was observed in five isolates, with four strains (*E. hormaechei* ERA1, *E. cloacae* ERA9, *E. cloacae* ESOA, and *B. proteolyticus* ESOB2) maintaining activity even under high temperature and salinity conditions. Eight bacterial strains produced indole-3-acetic acid (IAA), but only *E. cloacae* ESOA, *B. stercoris* ERAS3, and *E. hormaechei* ERA1 remained efficient under stress, with IAA production ranging from 1.05 to 218.42 μg mL^−1^ (e.g*.*,* B. stercoris* ERAS3 under 50 mM NaCl and 37 °C)(Fig. 4a). Furthermore, ammonia production, used as an indirect indicator of nitrogenase activity, was observed in five strains (*R. aquimaris* ERAS4, *B. stercoris* ERAS3, *B. cabrialesii* ERAS1, *S. marcescens* ERA6, and *B. paramycoides* ERA3) as shown in Fig. 4b.

The characterization identified five top-performing strains based on their combined PGP traits:

- ERA1: biofilm formation, phosphate solubilization, and IAA synthesis
–ERA6: biofilm formation, ammonia production, and protease secretion
–ERA9: biofilm formation, phosphate solubilization, and antioxidant activity
–ESOA: swarming, ammonia production, and IAA synthesis
- ESOB2: swarming, ammonia production, and antioxidant activity

No single strain displayed all tested PGP activities under increasing stress, prompting the formulation of bacterial consortia to combine complementary functionalities. Three consortia were formulated:

- CONSI: ERA1 + ESOA + ESOB2
- CONSII: ERA1 + ESOA + ERA6
- CONSIII: ERA6 + ERA9 + ESOB2

Compatibility tests confirmed that the following consortia could grow together without mutual inhibition.

### Effect of salt‐tolerant plant growth-promoting bacteria on wheat growth under salt and temperature stress

#### In vitro experiments

**Fig. 5:**
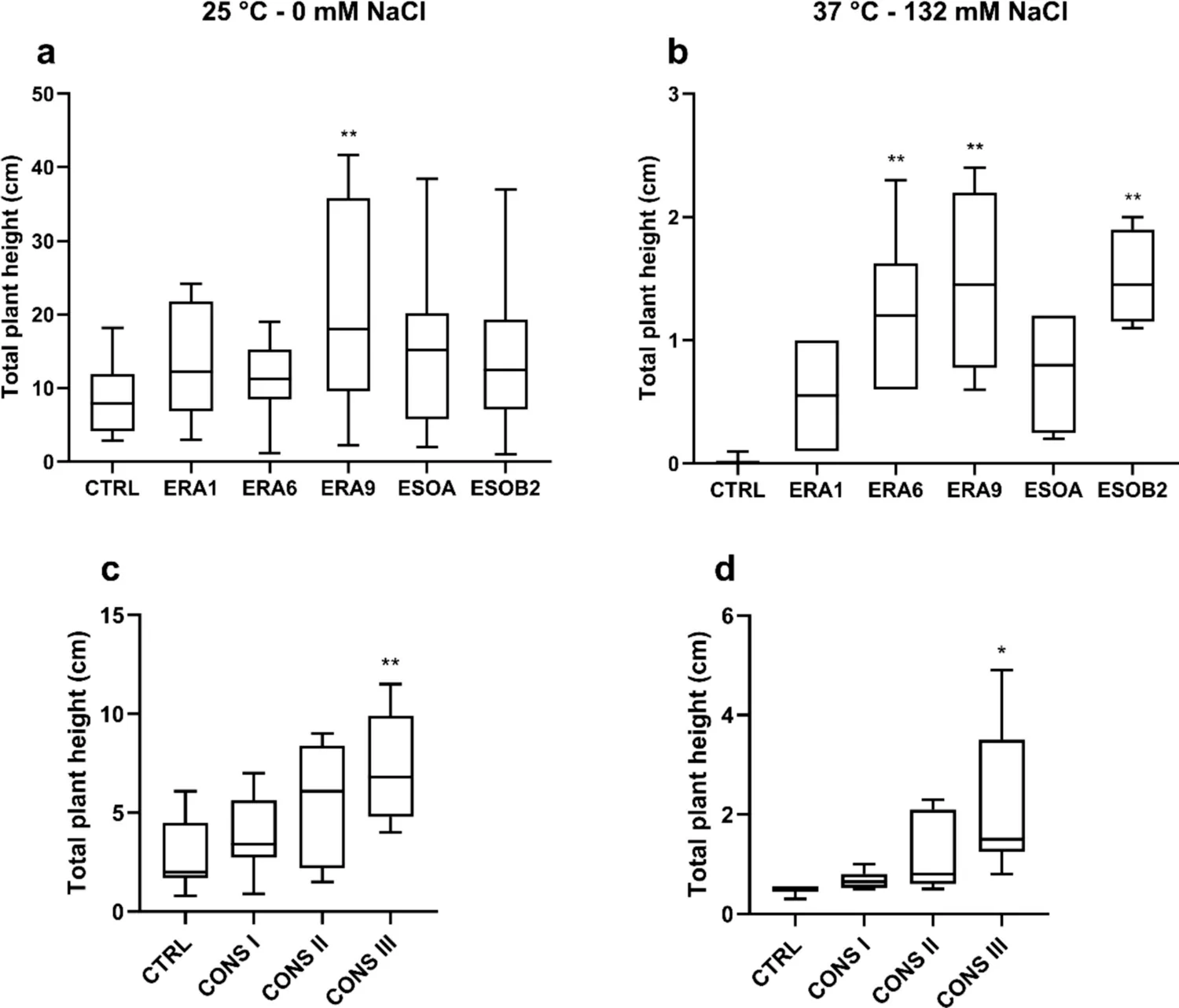
In vitro germination of wheat seedlings in the presence of PGPR. Wheat seedlings were incubated overnight with each strain or consortium and deposited on water-agar plates at 25 °C without salt (**a**, **c**) and at 37 °C with 132 mM of NaCl (**b**, **d**). The total plant length is represented as the sum of shoot and root length. Results are reported as mean (*n* = 24) ± SEM (standard error of the mean) of three independent experiments. A one-way ANOVA test was performed to compare the groups of data, and *p*-values are shown in the figure. Asterisks indicate significant differences with the control (CTRL) according to Tukey’s test, *p < 0.05; **p < 0.005

The five selected bacterial strains and the three consortia were tested on wheat (*T. durum *cv. Creso), a staple crop sensitive to environmental stresses, to assess their ability to promote plant growth under physiological (25 °C without salt) and stress conditions (37 °C with 132 mM NaCl) (Canton 2021; Upadhyay et al. 2012). Wheat seeds inoculated with bacterial strains (10^8^ cells mL^−1^) were grown on H₂O-agar plates, and growth was evaluated after 7 days by measuring the combined root and shoot lengths (Fig. 5).

Under physiological conditions, all bacterial strains enhanced wheat growth compared to the control, with *E. cloacae* ERA9 showing the most pronounced effect in plant height (Fig. 5a). Under stress, seed germination was completely inhibited without bacterial inoculation (Fig. 5b). However, the inoculation of bacterial strains restored the growth of wheat plants, with *E. cloacae* ERA9 once again standing out for producing the most beneficial effect on the plant. Interestingly, it primarily enhances root growth under physiological conditions, while it significantly boosts shoot growth under multi-stress conditions (Supplemental Table S4, Supplemental Fig. S2). Consortia testing revealed that CONSIII (composed of *S. marcescens* ERA6, *E. cloacae* ERA9, and *B. proteolyticus* ESOB2) significantly improved wheat growth in both physiological and stress conditions (Fig. 5c, d, Supplemental Table S5, Supplemental Fig. S3). CONSIII increased plant length by 60.3% under physiological conditions and 81.0% under stress. This synergistic effect likely stems from the complementary PGP traits of the constituent strains, including phosphate solubilization, ammonia production, and antioxidant activity. In contrast, CONSI and CONSII showed limited or moderate growth promotion and were less effective than CONSIII; for this reason, only CONSIII was used in subsequent experiments.

#### Pot experiments

**Fig. 6:**
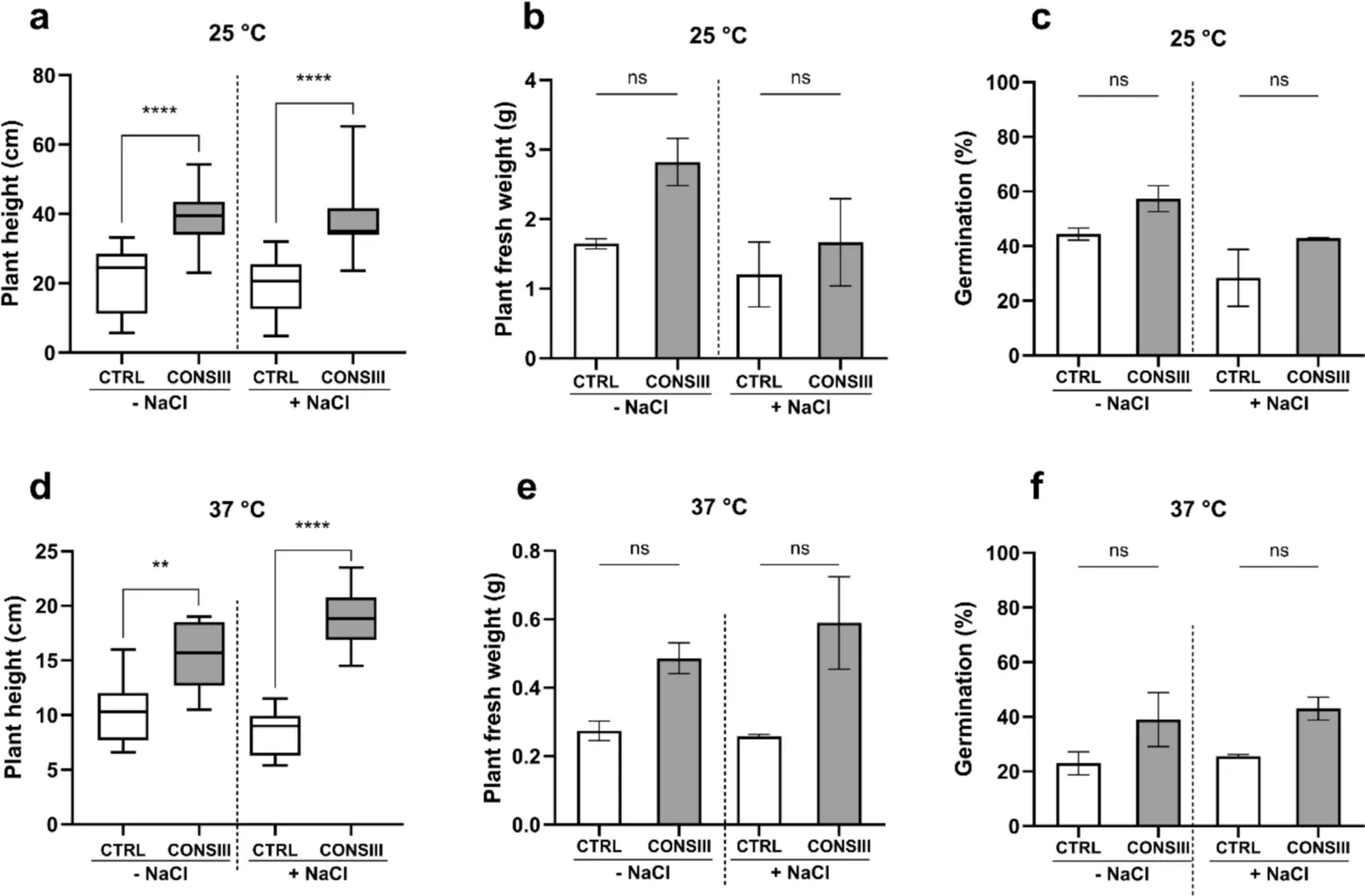
Effect of CONSIII on morphological characteristics of wheat plants under different salt concentrations and temperatures (**a**, **b**, **c** 25 °C; **d**, **e**, **f** 37 °C). Wheat seeds were incubated overnight with CONSIII and sown in sterile soil-filled pots and then incubated at the respective temperatures with a 16 h:8 h, light/night cycle. The total plant height (**a** and **d**) is represented as the sum of shoot and root length. Results are reported as mean (*n* = 28) ± SEM (standard error of the mean) of two independent experiments. Statistical analysis was performed using an unpaired *t-test*, comparing each treatment with CONSIII with the respective control without bacteria (CTRL). Statistical significance is reported with asterisks on the graphs; ns, not significant; **p < 0.01; ****p < 0.0001

**Fig. 7:**
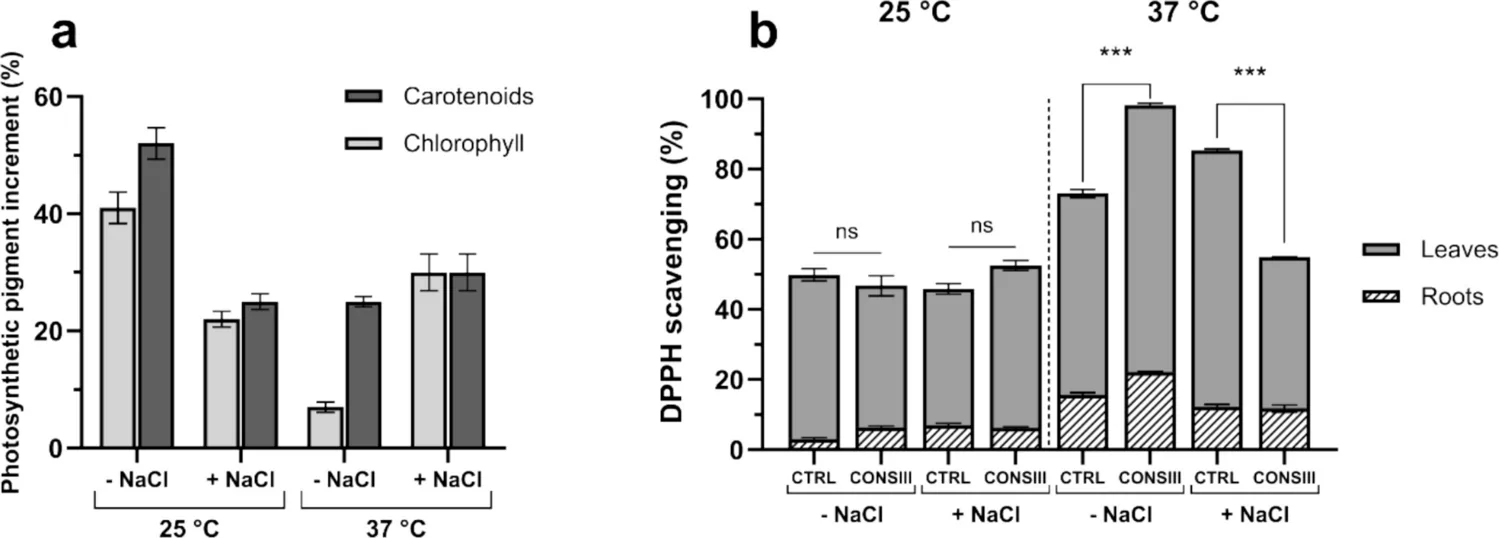
Effect of CONSIII on biochemical markers of wheat plants under different salt concentrations and temperatures. Chlorophyll and carotenoid (**a**) contents are reported as mg of pigment per gram of fresh weight (FW). The antioxidant activity is reported as a percentage of 2,2-diphenyl-1-picrylhydrazyl (DPPH) radical scavenging activity observed in leaves and roots (**b**). Results are reported as mean ± SEM (standard error of the mean) (*n* = 5). Statistical analysis was performed using an unpaired *t-test*, comparing each treatment with CONSIII with the respective control without bacteria (CTRL). Statistically significant results are reported with asterisks on the graphs; ns, not significant; **p < 0.01; ****p < 0.0001

Pot trials further validated the in vitro findings under more realistic conditions. Wheat plants were grown in pots under both physiological conditions and double abiotic stress conditions (37 °C with 132 mM NaCl). Growth and some biochemical markers were measured after 21 days from germination, and the results are reported in Figs. 6 and 7, respectively.

Wheat plants treated with CONSIII exhibited significant growth improvement under both non-stress and stress conditions, particularly in plant height. As shown in Fig. 6a, wheat plants treated with CONSIII at 25 °C exhibited an 82% increase in height under normal conditions and a 95% increase under salt stress. Similarly, at 37 °C (Fig. 6d), CONSIII enhanced plant height by 49% in the absence of salt stress and by 122% under osmotic stress, increasing from 8.48 cm in the control to 18.86 cm. Even if no statistically significant differences were observed in terms of germination rate and plant fresh weight (Fig. 6b, e, c, f), a positive trend was observed, confirming the increase in plant length treated with CONSIII and reinforcing the synergistic effect of the three PGPR strains in promoting wheat growth and improving stress resilience in a more natural setting. Biochemical analyses indicate that temperature influences chlorophyll and carotenoid production, as shown in Fig. 7a.

As shown in Fig. 7a, carotenoids and chlorophyll levels increase in all the conditions tested compared to the control, especially in the physiological conditions (25 °C without salt stress). Conversely, in Fig. 7b, the antioxidant activity of roots and leaves is strongly affected by temperature and salt concentration, while there are no significant differences at 25 °C among the samples. These findings lead to further interpretation of how bacterial inoculation modulates plant physiological and biochemical responses under stress, as discussed below.

## Discussion

This work highlights the potential of halotolerant PGPR strains isolated from *P. maritimum* roots to improve wheat growth under combined stress, high temperature and salinity. Through selective enrichment, 15 bacterial strains both halo- and thermotolerant have been isolated and identified, with the majority of them belonging to the genera of *Bacillus*,* Pseudomonas*, and *Enterobacter*, commonly associated with stress-resilient PGPR in saline and arid soils. When tested for their PGP activities, five bacterial strains (*B. proteolyticus* ESOB2, *B. stercoris* ERAS3, *B. cabrialesii* ERAS1, *B. paramycoides* ERA3, and *S. marcescens* ERA6) revealed a strong hydrolytic activity. Commonly, PGPR possess the ability to enhance plants’ rhizosphere environment by modulating soil enzyme activity and improving its fertility. The enzymatic activities of PGPR in the rhizosphere thus represent a valuable indicator for assessing soil stress levels (del Carmen Rivera-Cruz et al. 2008). Several studies have shown that many rhizobacteria can synthesize extracellular hydrolytic enzymes that are involved in breaking down complex macromolecules, thus degrading them into simpler compounds, thereby accelerating nutrient mineralization and indirectly increasing the availability of essential nutrients for plant uptake (Pang et al. 2004; Shaikh and Sayyed 2014). However, despite some progress in understanding the effects of soil salinity on enzyme activity (Pan et al. 2020), few studies have focused on the enzymatic responses of PGPR under combined temperature and salinity stress conditions (Pal et al. 2006; Ren et al. 2021). Our study fills this gap by evaluating the hydrolytic potential of PGPR strains under dual stress conditions. The results highlight the remarkable resilience and enzymatic versatility of the isolates, able to produce robust amylase, cellulase, and protease, active even under combined high-temperature and salinity stress. This characteristic is likely linked to their origin in the rhizosphere of *P. maritimum*, a coastal plant exposed to an environment characterized by high salinity, wide temperature variations, and nutrient scarcity. Under such selective pressures, the associated rhizosphere may have developed enzymes with exceptional stability and catalytic efficiency to ensure nutrient turnover and support plant growth under otherwise limiting conditions. This finding underscores the high potential of extreme or marginal ecosystems as a source of stress-resistant microbial strains and the usefulness of applying enrichment strategies to select higher-performing PGPR candidates. The production of hydrolytic enzymes not only contributes to nutrient acquisition but also holds potential antifungal properties by targeting the cell walls of phytopathogenic fungi, as shown by the inhibition of *P. nodorum* and *P. tritici-repentis*, two major fungal pathogens of wheat, by *B. proteolyticus* ESOB2 and *B. stercoris* ERAS3 and mainly by *B. cabrialesii* ERAS1. Recent genomic study on *B. cabrialesii* revealed the presence of several biosynthetic gene clusters (BGCs) for lipopeptides and other secondary metabolites with antifungal activity, such as surfactine, fengicine, and bacillaene (Valenzuela-Aragon et al. 2024). The microscopic alterations in the fungal hyphae, observed when the pathogens are co-cultured with *B. cabrialesii* ERAS1, may be the result of a synergistic effect between the enzymatic degradation of fungal cell walls and the secretion of diffusible antifungal compounds. In addition, the swellings visible in the fungal cells suggest a potential autophagosome-like structure (Li et al. 2014). Further experiments should be carried out to confirm the hypothesis and establish the mechanisms of fungal inhibition. This antagonistic capacity reinforces the multifunctionality of the isolated strains, which can enhance both plant nutrition and health simultaneously, consistent with reports describing the dual role of *Bacillus*-derived enzymes in nutrient cycling and pathogen suppression (Pal et al. 2006; Ren et al. 2021).

In addition to their enzymatic and antifungal properties, the 15 bacterial isolates displayed different PGP properties, which were stable under the selected multi-stress conditions. Traits such as swarming motility and biofilm formation are important for root surface colonization and adherence, intracellular communication, and long-term survival in the rhizosphere during stress conditions. Formation of biofilm, particularly, enhances osmotic and oxidative stress resistance in bacteria by stimulating the production of extracellular polymeric substances, which protect the cells from dehydration and ionic imbalance (Kasim et al. 2016). Moreover, IAA production, stable also under combined stress, can promote root elongation and nutrient uptake under adverse conditions, while ammonia provides a readily available nitrogen source that sustains chlorophyll synthesis and growth during salinity and heat stress (Spaepen et al. 2007). Due to the different traits of the strains, consortia were assembled to enhance the PGP characteristics of the single strains. When tested on wheat plants, the CONSIII, including *S. marcescens* ERA6, *E. cloacae* ERA9, and *B. proteolyticus* ESOB2, emerged as the best performer under all experimental conditions. The beneficial effects observed in in vitro and in pot experiments may be due to the complementary functions of its bacterial members. Performed analysis (Table S6) revealed complementary PGP traits of the three strains, including protease, cellulase, and chitinase activity, high IAA and NH_4_ production efficiency, phosphate solubilization, and significant antioxidant activity even at 37 °C and in the presence of salt. Recent study has established that *S. marcescens* can induce wheat growth under salinity through ACC (1-aminocyclopropane-1-carboxylic acid) deaminase activity, siderophore production, and phosphate solubilization, inhibiting ethylene synthesis and promoting root elongation (Singh and Jha 2016). Similarly, *E. cloacae* subsp. *dissolvens* strains with ACC deaminase activity have been found to modulate wheat stress proteins and nutrient uptake under high NaCl (Singh and Sharma, 2017). *B. proteolyticus*, recently reported to be salt-tolerant PGPB, can produce IAA and gibberellins, phosphorus solubilization, and stimulate wheat shoot and root growth even under 500 mM NaCl (Metoui Ben Mahmoud et al. 2020). Therefore, the simultaneous presence of these taxa in CONSIII can give rise to a multi-faceted protection strategy: hormonal regulation (IAA, gibberellins, ACC deaminase) facilitating root growth; enzymatic degradation of organic matter facilitating nutrient assimilation; and antioxidant defence lowering oxidative and ionic stress. Interestingly, the CONSIII demonstrates a more effective response than the control (CTRL) in inducing a significant increase in carotenoids at 37 °C, suggesting that the presence of bacteria reduces plant stress, thereby supporting light harvesting, photosynthesis, and plant growth even under unfavourable temperature conditions. The enhanced antioxidant activity at higher temperatures, mainly in roots, could be due to the observed increase of carotenoids, which are known to play an active role in scavenging processes related to oxidative stress and reactive oxygen species (ROS) removal (Zhang et al. 2012). Conversely, in roots, the absence of pigments may contribute to explaining the different response of antioxidant activity compared to leaves. Indeed, roots being more exposed to salt stress in the cultivation mean likely need to recruit higher antioxidant defences (Bandeoğlu et al. 2004). Notably, while previous studies report either salinity or temperature stress individually, our research presents the evidence that a carefully selected microbial consortia can enhance wheat resistance to both heat and salinity simultaneously, thus representing a promising tool for sustainable agriculture under changing climatic conditions.

## Conclusions

The cumulative impact of human life on our planet over the past few decades has led to the emergence of many extreme environmental conditions affecting ecosystems and agricultural land. Global warming, climate change, and pollution expose plants to unique combinations of multiple abiotic and biotic stresses simultaneously. This study highlights the potential of PGPR isolated from *P. maritimum* in promoting the growth of plants and their resilience under abiotic stress conditions, like salinity and high temperature. Using an enrichment approach, a core group of halotolerant and thermotolerant PGPR capable of maintaining multiple PGP functions under abiotic stress conditions was isolated. Five top-performing strains were combined in three consortia and tested for their ability to promote wheat growth under physiological and double abiotic stress conditions. This study clearly demonstrates the importance of PGPR in mitigating the harmful consequences of abiotic stress on plants. The results observed with CONSIII-treated plants suggest that bacterial inoculation can sustain plant growth and photosynthesis under adverse conditions, counteracting the accumulation of ROS and supporting overall plant health. These benefits are probably due to complementary PGP traits of CONSIII, lying in the fact that phosphate solubilization, ammonia production, and antioxidant activity act together to promote plant growth and stress tolerance. In conclusion, CONSIII represents a promising microbial consortium for developing sustainable agricultural solutions to improve crop productivity under challenging environmental conditions.

## Supplementary information

Below is the link to the electronic supplementary material.

ESM 1 (DOCX 1.23 MB)

## Data Availability

Sequence data that support the findings of this study have been deposited in the National Center for Biotechnology Information (NCBI) Sequence Read Archive. The accession numbers of 16S RNA sequences are provided in the result section (Table 1).

## References

[CR1] Alizadeh MR, Adamowski J, Nikoo MR, AghaKouchak A, Dennison P, Sadegh M (2020) A century of observations reveals increasing likelihood of continental-scale compound dry-hot extremes. Sci Adv 6:eaaz4571. 10.1126/sciadv.aaz4571 32967839 10.1126/sciadv.aaz4571 PMC7531886

[CR2] Bandeoğlu E, Eyidoğan F, Yücel M, Avni Öktem H (2004) Antioxidant responses of shoots and roots of lentil to NaCl-salinity stress. Plant Growth Regul 42:69–77. 10.1023/B:GROW.0000014891.35427.7b

[CR3] Barbulova A, D’Apuzzo E, Rogato A, Chiurazzi M (2005) Improved procedures for in vitro regeneration and for phenotypic analysis in the model legume *Lotus japonicus*. Funct Plant Biol 32:529–536. 10.1071/FP05015 32689153 10.1071/FP05015

[CR4] Brouder SM, Volenec JJ (2008) Impact of climate change on crop nutrient and water use efficiencies. Physiol Plant 133:705–724. 10.1111/j.1399-3054.2008.01136.x 18507815 10.1111/j.1399-3054.2008.01136.x

[CR5] Canton H (2021) Food and Agriculture Organization of the United Nations — FAO, twenty-third ed. Routledge

[CR6] Carmona MA, Ferrazini M, Barreto DE (2006) Tan spot of wheat caused by *Drechslera tritici-repentis*: detection, transmission, and control in wheat seed. Cereal Res Commun 34:1043–1049. 10.1556/CRC.34.2006.2-3.236

[CR7] Castaldi S, Lorenz C, Vitale E, Santorufo L, Isticato R, Arena C (2024) Potentialities of technosol-isolated PGPB consortium in promoting plant growth in lettuce seedlings. Plant Soil 507:475–495. 10.1007/s11104-024-06746-z

[CR8] Castaldi S, Zorrilla JG, Petrillo C, Russo MT, Ambrosino P, Masi M, Cimmino A, Isticato R (2023) *Alternaria alternata* isolated from infected pears (*Pyrus communis*) in Italy produces non-host toxins and hydrolytic enzymes as infection mechanisms and exhibits competitive exclusion against *Botrytis cinerea* in co-infected host fruits. J Fungi 9(3):326. 10.3390/jof9030326 10.3390/jof9030326 PMC10053571 36983494

[CR9] Chakraborty S, Tiedemann AV, Teng PS (2000) Climate change: potential impact on plant diseases. Environ Pollut 108:317–326. 10.1016/S0269-7491(99)00210-9 15092926 10.1016/s0269-7491(99)00210-9

[CR10] Chaves MM, Maroco JP, Pereira JS (2003) Understanding plant responses to drought - from genes to the whole plant. Funct Plant Biol 30:239–264. 10.1071/FP02076 32689007 10.1071/FP02076

[CR11] Coolen S, Proietti S, Hickman R, Davila Olivas NH, Huang P, Van Verk MC, Van Pelt JA, Wittenberg AHJ, De Vos M, Prins M (2016) Transcriptome dynamics of *Arabidopsis* during sequential biotic and abiotic stresses. Plant J 86:249–267. 10.1111/tpj.13167 26991768 10.1111/tpj.13167

[CR12] Defo MA, Gendron AD, Head J, Pilote M, Turcotte P, Marcogliese DJ, Houde M (2019) Cumulative effects of cadmium and natural stressors (temperature and parasite infection) on molecular and biochemical responses of juvenile rainbow trout. Aquat Toxicol 217:105347. 10.1016/j.aquatox.2019.105347 31715476 10.1016/j.aquatox.2019.105347

[CR13] del Carmen R-C, Narcía AT, Ballona GC, Kohler J, Caravaca F, Roldan A (2008) Poultry manure and banana waste are effective biofertilizer carriers for promoting plant growth and soil sustainability in banana crops. Soil Biol Biochem 40:3092–3095. 10.1016/j.soilbio.2008.09.003

[CR14] Demutskaya L, Kalinichenko I (2010) Photometric determination of ammonium nitrogen with the Nessler reagent in drinking water after its chlorination. J Water Chem Technol 32:90–94. 10.3103/S1063455X10020049

[CR15] Downie RC, Lin M, Corsi B, Ficke A, Lillemo M, Oliver RP, Phan HTT, Tan KC, Cockram J (2021) *Septoria nodorum* blotch of wheat: disease management and resistance breeding in the face of shifting disease dynamics and a changing environment. Phytopathology 111:906–920. 10.1094/PHYTO-07-20-0280-RVW 33245254 10.1094/PHYTO-07-20-0280-RVW

[CR16] Gordon SA, Weber RP (1951) Colorimetric estimation of indoleacetic acid. Plant Physiol 26(1):192–195. 10.1104/pp.26.1.192 16654351 10.1104/pp.26.1.192 PMC437633

[CR17] Grimm NB, Foster D, Groffman P, Grove JM, Hopkinson CS, Nadelhoffer KJ, Pataki DE, Peters DPC (2008) The changing landscape: ecosystem responses to urbanization and pollution across climatic and societal gradients. Front Ecol Environ 6:264–272. 10.1890/070147

[CR18] Guo J, Shi G, Kalil A, Friskop A, Elias E, Xu SS, Faris JD, Liu Z (2020) *Pyrenophora tritici-repentis* race 4 isolates cause disease on tetraploid wheat. Phytopathol 110:1781–1790. 10.1094/PHYTO-05-20-0179-R 10.1094/PHYTO-05-20-0179-R 32567977

[CR19] Hafez M, Gourlie R, McDonald M, Telfer M, Carmona MA, Sautua FJ, Moffat CS, Moolhuijzen PM, See PT, Aboukhaddour R (2023) Evolution of the *toxB* gene in *Pyrenophora tritici-repentis* and related species. Mol Plant-Microbe Interact 37:327–337. 10.1094/MPMI-08-23-0114-FI 10.1094/MPMI-08-23-0114-FI 37759383

[CR20] Haiyambo DH, Chimwamurombe PM, Reinhold-Hurek B (2015) Isolation and screening of rhizosphere bacteria from grasses in east Kavango region of Namibia for plant growth promoting characteristics. Curr Microbiol 71:566–571. 10.1007/s00284-015-0886-7 26254764 10.1007/s00284-015-0886-7

[CR21] Hamann E, Blevins C, Franks SJ, Jameel MI, Anderson JT (2021) Climate change alters plant–herbivore interactions. New Phytol 229:1894–1910. 10.1111/nph.17036 33111316 10.1111/nph.17036

[CR22] Hankin L, Anagnostakis SL (1977) Solid media containing carboxymethylcellulose to detect CX cellulase activity of micro-organisms. Microbiology 98:109–115. 10.1099/00221287-98-1-109 10.1099/00221287-98-1-109 401863

[CR23] Kai H, Iba K (2014) Temperature stress in plants. In: eLS. John Wiley & Sons, Ltd, Chichester. 10.1002/9780470015902.a0001320.pub2 I

[CR24] Kallenborn R, Halsall C, Dellong M, Carlsson P (2012) The influence of climate change on the global distribution and fate processes of anthropogenic persistent organic pollutants. J Environ Monit 14:2854–2869. 10.1039/C2EM30519D 23014859 10.1039/c2em30519d

[CR25] Kasim WA, Gaafar RM, Abou-Ali RM, Omar MN, Hewait HM (2016) Effect of biofilm forming plant growth promoting rhizobacteria on salinity tolerance in barley. Ann Agric Sci 61:217–227. 10.1016/j.aoas.2016.07.003

[CR26] Khamna S, Yokota A, Lumyong S (2009) *Actinomycetes* isolated from medicinal plant rhizosphere soils: diversity and screening of antifungal compounds, indole-3-acetic acid and siderophore production. World J Microbiol Biotechnol 25:649–655. 10.1007/s11274-008-9933-x

[CR27] Kumar S, Stecher G, Suleski M, Sanderford M, Sharma S, Tamura K (2024) MEGA12: molecular evolutionary genetic analysis version 12 for adaptive and green computing. Mol Biol Evol 41(12):msae263. 10.1093/molbev/msae263 39708372 10.1093/molbev/msae263 PMC11683415

[CR28] Lee H, Calvin K, Dasgupta D, Krinner G, Mukherji A, Thorne P, Trisos C, Romero J, Aldunce P, Barret K (2023) Climate change 2023: synthesis report, summary for policymakers. In: Core Writing Team, Lee H and Romero J (eds) IPCC, 2023. Accessed 16 Dec 2025. https://www.ipcc.ch/report/ar6/syr/downloads/report/IPCC_AR6_SYR_SPM.pdf

[CR29] Lee JS, Bae YM, Han A, Lee SY (2016) Development of Congo red broth method for the detection of biofilm-forming or slime-producing *Staphylococcus* sp. LWT 73:707–714. 10.1016/j.lwt.2016.03.023

[CR30] Li H, Zhang S, Lu J, Liu L, Uluko H, Pang X, Sun Y, Xue H, Zhao L, Kong F, Lv J (2014) Antifungal activities and effect of *Lactobacillus casei* AST18 on the mycelia morphology and ultrastructure of *Penicillium chrysogenum*. Food Control 43:57–64. 10.1016/j.foodcont.2014.02.045

[CR31] Lichtenthaler HK (1987) Chlorophylls and carotenoids: Pigments of photosynthetic biomembranes. In: Packer L, Douce R (eds) Plant cell membranes. Academic Press, Meth Enzymol 148:350–382. 10.1016/0076-6879(87)48036-1

[CR32] Lindemann SR, Bernstein HC, Song HS, Fredrickson JK, Fields MW, Shou W, Johnson DR, Beliaev AS (2016) Engineering microbial consortia for controllable outputs. ISME J 10:2077–2084. 10.1038/ismej.2016.26 26967105 10.1038/ismej.2016.26 PMC4989317

[CR33] McKenzie RL, Aucamp PJ, Bais AF, Björn LO, Ilyas M, Madronich S (2011) Ozone depletion and climate change: impacts on UV radiation. Photochem Photobiol Sci 10:182–198. 10.1039/C0PP90034F 21253660 10.1039/c0pp90034f

[CR34] Meddeb-Mouelhi F, Moisan JK, Beauregard M (2014) A comparison of plate assay methods for detecting extracellular cellulase and xylanase activity. Enzyme Microb Technol 66:16–19. 10.1016/j.enzmictec.2014.07.004 25248694 10.1016/j.enzmictec.2014.07.004

[CR35] Metoui Ben Mahmoud O, Hidri R, Talbi-Zribi O, Taamalli W, Abdelly C, Djébali N (2020) Auxin and proline producing rhizobacteria mitigate salt-induced growth inhibition of barley plants by enhancing water and nutrient status. South Afr J Bot 128:209–217. 10.1016/j.sajb.2019.10.023

[CR36] Morris LS, Evans J, Marchesi JR (2012) A robust plate assay for detection of extracellular microbial protease activity in metagenomic screens and pure cultures. J Microbiol Methods 91:144–146. 10.1016/j.mimet.2012.08.006 22921428 10.1016/j.mimet.2012.08.006

[CR37] Mosallanejad N, Zarei M, Ghasemi-Fasaei R, Shahriari AG, Mohkami A, Janda T (2025) Effect of *Claroideoglomus etunicatum* and indole-3-acetic acid on growth and biochemical properties of vetiver grass (*Vetiveria zizanioides*) under salinity stress. Int J Mol Sci 26(7):3132. 10.3390/ijms26073132 40243920 10.3390/ijms26073132 PMC11989100

[CR38] Naylor D, Coleman-Derr D (2018) Drought stress and root-associated bacterial communities. Front Plant Sci 8:2223. 10.3389/fpls.2017.02223 29375600 10.3389/fpls.2017.02223 PMC5767233

[CR39] Nicholson WL, Setlow P (1990) Sporulation, germination and outgrowth. In: Harwood CR, Cutting SM (eds). (1990). Molecular biological methods for *Bacillus*. John Wiley, Chichester, UK pp 391–450

[CR40] Nimisha P, Moksha S, Gangawane AK (2019) Amylase activity of starch degrading bacteria isolated from soil. Int J Curr Microbiol Appl Sci 8:659–671. 10.20546/ijcmas.2019.804.071

[CR41] Pal KK, Scholar V, Gardener BM (2006) Biological control of plant pathogens. Plant Health Instr 2:1117–1142. 10.1094/PHI-A-2006-1117-02

[CR42] Pan J, Huang C, Peng F, Zhang W, Luo J, Ma S, Xue X (2020) Effect of arbuscular mycorrhizal fungi (AMF) and plant growth-promoting bacteria (PGPR) inoculations on *Elaeagnus angustifolia* L. in saline soil. Appl Sci 10:945. 10.3390/app10030945

[CR43] Pang Z, Otaka K, Suzuki Y (2004) Purification and characterization of an endo-1, 3-β-glucanase from *Arthrobacter* sp. J Biol Macromol 4:57–66

[CR44] Petrillo C, Vitale E, Ambrosino P, Arena C, Isticato R (2022) Plant growth-promoting bacterial consortia as a strategy to alleviate drought stress in *Spinacia oleracea*. Microorganisms 10(9):1798. 10.3390/microorganisms10091798 36144400 10.3390/microorganisms10091798 PMC9501077

[CR45] Ragucci S, Castaldi S, Landi N, Isticato R, Di Maro A (2023) Antifungal activity of ageritin, a ribotoxin-like protein from *Cyclocybe aegerita* edible mushroom, against phytopathogenic fungi. Toxins 15(9):578. 10.3390/toxins15090578 37756004 10.3390/toxins15090578 PMC10535218

[CR46] Ren H, Lv C, Fernández-García V, Huang B, Yao J, Ding W (2021) Biochar and PGPR amendments influence soil enzyme activities and nutrient concentrations in a eucalyptus seedling plantation. Biomass Conv Bioref 11:1865–1874. 10.1007/s13399-019-00571-6

[CR47] Rosenzweig C, Hillel D (2000) Soils and global climate change: challenges and opportunities. Soil Sci 165(1):47–56. 10.1097/00010694-200001000-00007

[CR48] Schoebitz M, Ceballos C, Ciamp L (2013) Effect of immobilized phosphate solubilizing bacteria on wheat growth and phosphate uptake. J Soil Sci Plant Nutr 13:1–10. 10.4067/S0718-95162013005000001

[CR49] Semwal P, Dave A, Israr J, Misra S, Kumar M, Paul D (2025) Exploring microbial ecosystem services for environmental stress amelioration: a review. Int J Mol Sci 26(10):4515. 10.3390/ijms26104515 40429660 10.3390/ijms26104515 PMC12111249

[CR50] Shaikh SS, Sayyed RZ (2014) Role of plant growth-promoting rhizobacteria and their formulation in biocontrol of plant diseases. In: Arora N (ed) Plant microbes symbiosis: Applied facets. Springer, pp 337–351. 10.1007/978-81-322-2068-8_18

[CR51] Shilev S (2020) Plant-growth-promoting bacteria mitigating soil salinity stress in plants. Appl Sci 10:7326. 10.3390/app10207326

[CR52] Silletti S, Di Stasio E, Van Oosten MJ, Ventorino V, Pepe O, Napolitano M, Marra R, Woo SL, Cirillo V, Maggio A (2021) Biostimulant activity of *Azotobacter chroococcum* and *Trichoderma harzianum* in durum wheat under water and nitrogen deficiency. Agronomy 11(2):380. 10.3390/agronomy11020380

[CR53] Singh N, Sharma DP, Kaushal R (2017) Effect of different rootstocks and soil agro-techniques on enzymatic activities, rhizospheric microbial counts and growth traits on apple replant sick soil. Pharma Innov J 6(12):288–293

[CR54] Singh RP, Jha PN (2016) The multifarious PGPR *Serratia marcescens* CDP-13 augments induced systemic resistance and enhanced salinity tolerance of wheat (*Triticum aestivum* L.). PLoS ONE 11(6):e0155026. 10.1371/journal.pone.0155026 27322827 10.1371/journal.pone.0155026 PMC4913913

[CR55] Spaepen S, Vanderleyden J, Remans R (2007) Indole-3-acetic acid in microbial and microorganism-plant signaling. FEMS Microbiol Rev 31:425–448. 10.1111/j.1574-6976.2007.00072.x 17509086 10.1111/j.1574-6976.2007.00072.x

[CR56] Stucky BJ (2012) Seqtrace: a graphical tool for rapidly processing DNA sequencing chromatograms. J Biomol Tech 23:90–93. 10.7171/jbt.12-2303-004 22942788 10.7171/jbt.12-2303-004 PMC3413935

[CR57] Suzuki N, Bassil E, Hamilton JS, Inupakutika MA, Zandalinas SI, Tripathy D, Luo Y, Dion E, Fukui G, Kumazaki A (2016) ABA is required for plant acclimation to a combination of salt and heat stress. PLoS ONE 11(1):e0147625. 10.1371/journal.pone.0147625 26824246 10.1371/journal.pone.0147625 PMC4733103

[CR58] Tejedor E, Benito G, Serrano-Notivoli R, González-Rouco F, Esper J, Büntgen U (2024) Recent heatwaves as a prelude to climate extremes in the western Mediterranean region. Npj Clim Atmos Sci 7:218. 10.1038/s41612-024-00771-6

[CR59] Teuling AJ (2018) A hot future for European droughts. Nat Clim Change 8:364–365. 10.1038/s41558-018-0154-5

[CR60] Tsavkelova EA, Klimova SI, Cherdyntseva TA, Netrusov AI (2006) Microbial producers of plant growth stimulators and their practical use: a review. Appl Biochem Microbiol 42:117–126. 10.1134/S0003683806020013 16761564

[CR61] Umesha S, Singh PK, Singh RP (2018) Microbial biotechnology and sustainable agriculture. In: Singh RL, Mondal S (eds) Biotechnology for sustainable agriculture. Woodhead Publishing, Cambridge, pp. 185–205. 10.1016/B978-0-12-812160-3.00006-4

[CR62] Upadhyay SK, Singh JS, Saxena AK, Singh DP (2012) Impact of PGPR inoculation on growth and antioxidant status of wheat under saline conditions. Plant Biol 14:605–611. 10.1111/j.1438-8677.2011.00533.x 22136617 10.1111/j.1438-8677.2011.00533.x

[CR63] USDA Natural Resources Conservation Service (NRCS) (2014) Soil Electrical Conductivity. USDA. Accessed 16 Dec 2025. https://www.nrcs.usda.gov/sites/default/files/2022-10/Soil%20Electrical%20Conductivity.pdf

[CR64] Valenzuela-Aragon B, Montoya-Martínez AC, Parra-Cota FI, de los Santos-Villalobos S (2024) Genomic insight into a potential biological control agent for *Fusarium*-related diseases in potatoes: *Bacillus cabrialesii* subsp. *cabrialesii* strain PE1. Horticulturae 10(4):357. 10.3390/horticulturae10040357

[CR65] van Zelm E, Zhang Y, Testerink C (2020) Salt tolerance mechanisms of plants. Annu Rev Plant Biol 71:403–433. 10.1146/annurev-arplant-050718-100005 32167791 10.1146/annurev-arplant-050718-100005

[CR66] Vasseur-Coronado M, du Boulois HD, Pertot I, Puopolo G (2021) Selection of plant growth promoting rhizobacteria sharing suitable features to be commercially developed as biostimulant products. Microbiol Res 245:126672. 10.1016/j.micres.2020.126672 33418398 10.1016/j.micres.2020.126672

[CR67] Vittoria M, Saggese A, Di Gregorio Barletta G, Castaldi S, Isticato R, Baccigalupi L, Ricca E (2023a) Sporulation efficiency and spore quality in a human intestinal isolate of *Bacillus cereus*. Res Microbiol 174:104030. 10.1016/j.resmic.2023.104030 36738815 10.1016/j.resmic.2023.104030

[CR68] Vittoria M, Saggese A, Isticato R, Baccigalupi L, Ricca E (2023b) Probiotics as an alternative to antibiotics: genomic and physiological characterization of aerobic spore formers from the human intestine. Microorganisms. 10.3390/microorganisms11081978 37630538 10.3390/microorganisms11081978 PMC10458579

[CR69] Xiang H, Sun-waterhouse D, Cui C, Wang W, Dong K (2018) Modification of soy protein isolate by glutaminase for nanocomplexation with curcumin. Food Chem 268:504–512. 10.1016/j.foodchem.2018.06.059 30064791 10.1016/j.foodchem.2018.06.059

[CR70] Zandalinas SI, Balfagón D, Gómez-Cadenas A, Mittler R (2022) Plant responses to climate change: metabolic changes under combined abiotic stresses. J Exp Bot 73:3339–3354. 10.1093/jxb/erac073 35192700 10.1093/jxb/erac073

[CR71] Zhang Q, Wang L, Kong F, Deng Y, Li B, Meng Q (2012) Constitutive accumulation of zeaxanthin in tomato alleviates salt stress-induced photoinhibition and photooxidation. Physiol Plant 146:363–373. 10.1111/j.1399-3054.2012.01645.x 22578286 10.1111/j.1399-3054.2012.01645.x

[CR72] Zheng L, Ma X, Lang D, Zhang X, Zhou L, Wang L, Zhang X (2022) Encapsulation of *Bacillus pumilus* G5 from polyvinyl alcohol-sodium alginate (PVA-SA) and its implications in improving plant growth and soil fertility under drought and salt soil conditions. Int J Biol Macromol 209:231–243. 10.1016/j.ijbiomac.2022.04.017 35395281 10.1016/j.ijbiomac.2022.04.017

