# Supplementary material for: Plant–microbe synergy: employing coastal plant bacteria for wheat prosperity under combined saline and heat stress
Source: Appl Microbiol Biotechnol. 2025 Dec 24;109(1):285. doi: 10.1007/s00253-025-13678-w (PMC12740961; doi:10.1007/s00253-025-13678-w)
Supplement: Supplementary file 1 — (DOCX 1.23 MB) [file 253_2025_13678_MOESM1_ESM.docx]

**Applied Microbiology and Biotechnology**

**Supplemental Material**

**Plant-microbe Synergy: Employing Coastal Plant Bacteria for Wheat Prosperity under Combined Saline and Heat Stress**

**Ivana Staiano**^a^**, Stefany Castaldi**^a^**, Ermenegilda Vitale**^a^**, Carmen Arena**^a^**, Rachele Isticato**^a, b,c*^

^a^Department of Biology, University of Naples Federico II, Complesso Universitario Monte S. Angelo, Naples, Italy

^b^National Biodiversity Future Center (NBFC), Palermo 90133, Italy

^c^Interuniversity Center for Studies on Bioinspired Agro-Environmental Technology (BAT Center), Portici, NA, Italy

*Corresponding author at: Department of Biology, University of Naples Federico II, Complesso Universitario Monte S. Angelo, Naples, Italy. E-mail address: isticato@unina.it (R. Isticato).

**Table S1** Schematic representation of the set-up of the pot experiments on wheat plants without bacteria (CTRL) and in presence of the selected PGPR combined in a consortium (CONSIII).

| 25 °C | | | | |  | 37 °C | | | | |
| --- | --- | --- | --- | --- | --- | --- | --- | --- | --- | --- |
| 0 mM NaCl | |  | 132 mM NaCl | |  | 0 mM NaCl | |  | 132 mM NaCl | |
| CTRL | CONSIII |  | CTRL | CONSIII |  | CTRL | CONSIII |  | CTRL | CONSIII |

**Table S2** Preliminary characterization of bacterial isolates

| **Strain** | **Plant association** | **Salinity NaCl (mM)** | **Temperature range (°C)** | **Colony colour** | **Colony morphology** | **Spore formation** | **Swarming capacity** | **Biofilm formation** |
| --- | --- | --- | --- | --- | --- | --- | --- | --- |
| **ERA1** | Endophytic | 50-600 | 25-42 | Creamy-white | Circular | - | - | + |
| **ERA2** | Endophytic | 50-600 | 25-37 | Creamy-white | Flat | - | - | - |
| **ERA3** | Endophytic | 50-600 | 25-42 | Milky-white | Wrinkled | + | - | + |
| **ERA4** | Endophytic | 50-600 | 25-42 | Yellowish | Translucent | - | - | + |
| **ERA5** | Endophytic | 50-330 | 25-37 | Creamy-white | Circular | - | - | - |
| **ERA6** | Endophytic | 50-600 | 25-37 | Pinky-red | Circular | - | - | + |
| **ERA7** | Endophytic | 50-600 | 25-37 | Creamy-white | Circular | - | - | - |
| **ERA9** | Endophytic | 50-600 | 25-42 | Milky-white | Circular | - | - | + |
| **ESOA** | Exophytic | 50-330 | 25-42 | Creamy-white | Circular | - | + | + |
| **ESOB1** | Exophytic | 50-330 | 25-42 | Creamy-white | Circular | - | - | + |
| **ESOB2** | Exophytic | 50-330 | 25-42 | Milky-white | Irregular | + | + | - |
| **ERAS1** | Endophytic | 50-600 | 25-42 | Milky-white | Wrinkled | + | - | - |
| **ERAS2** | Endophytic | 50-330 | 25-37 | Creamy-white | Circular | - | - | - |
| **ERAS3** | Endophytic | 50-600 | 25-42 | Creamy-white | Circular | + | - | - |
| **ERAS4** | Endophytic | 50-600 | 25-37 | Orange | Wrinkled | + | - | - |

**Fig. S1** Phylogenetic analysis based on 16S rRNA gene sequences of bacterial isolates. The 16S rRNA sequence of *Aquifex aeolicus* (AJ309733.1) was used to assign an outgroup species

**
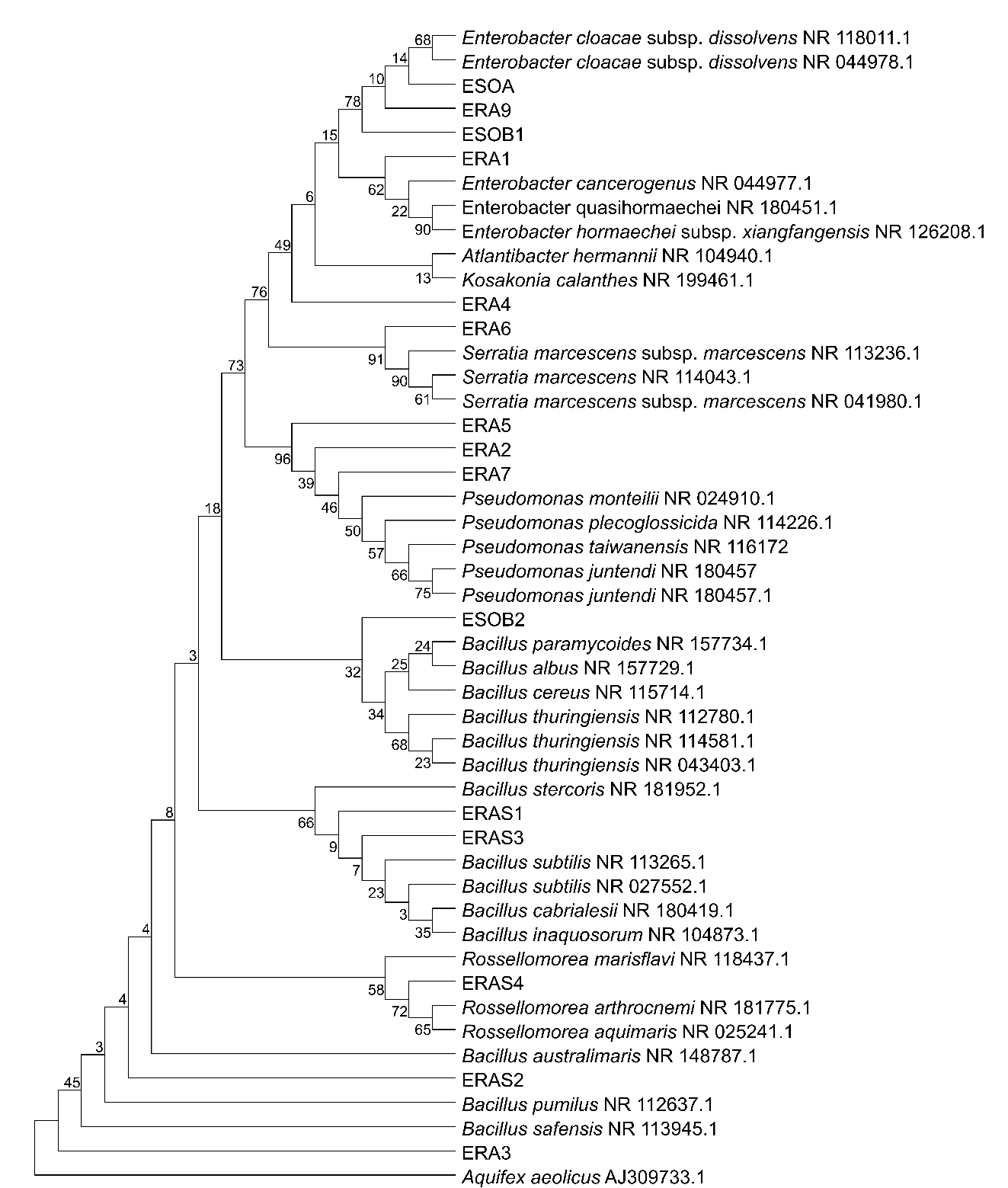
**

**Table S3** Phosphate solubilization of the top-performing strains evaluated by growing the bacteria on Pikovskaya medium. Results are reported as no activity (-), halo diameter < 5 mm (+), halo diameter ≥ 5 mm (++), experiment was conducted in triplicate. Strain ERAS2 is not able to grow at 42 °C, as reported above in Table S2

| **Strain ID** | **Temperature (°C)** | **PVK** | | |
| --- | --- | --- | --- | --- |
|  |  | 50 mM | 132 mM | 330 mM |
| ERA1 | 25 | ++ | + | + |
|  | 37 | ++ | + | + |
|  | 42 | + | - | + |
| ERA9 | 25 | + | ++ | ++ |
|  | 37 | ++ | ++ | ++ |
|  | 42 | + | + | ++ |
| ESOA | 25 | + | + | + |
|  | 37 | + | + | ++ |
|  | 42 | - | + | + |
| ESOB1 | 25 | ++ | ++ | ++ |
|  | 37 | ++ | ++ | ++ |
|  | 42 | ++ | + | + |
| ERAS2 | 25 | ++ | + | + |
|  | 37 | + | + | - |
|  | ~~42~~ |  |  |  |

**Table S4** In vitro growth of wheat seedlings in presence of single PGPR

|  | **25 °C – 0 mM NaCl** | | | | **37 °C – 132 mM NaCl** | | | |  |
| --- | --- | --- | --- | --- | --- | --- | --- | --- | --- |
| **Inoculation** | | **Root length (cm)** | **Shoot length (cm)** | **Total length (cm)** | | **Root length (cm)** | **Shoot length (cm)** | **Total length (cm)** | |
| CTRL | | 4.79 ± 0.83 | 3.69 ± 0.50 | 8.47 ± 1.23 | | 0.02 ± 0.01 | 0.01 ± 0.01 | 0.01 ± 0.01 | |
| ERA1 | | 10.64 ± 1.95 | 4.77 ± 1.18 | 13.02 ± 1.89 | | 0.23 ± 0.13 | 0.75 ± 0.25 | 0.55 ± 0.26 | |
| ERA6 | | 7.53 ± 1.02 | 4.96 ± 0.81 | 11.23 ± 1.39 | | 0.63 ± 0.15 | 0.56 ± 0.14 | 1.22 ± 0.26 | |
| ERA9 | | 12.06 ± 2.01 | 7.93 ± 1.19 | 19.99 ± 3.14 | | 0.63 ± 0.12 | 1.05 ± 0.25 | 1.48 ± 0.37 | |
| ESOA | | 8.14 ± 1.41 | 6.84 ± 1.21 | 14.97 ± 2.59 | | 0.43 ± 0.10 | 0.65 ± 0.15 | 0.75 ± 0.26 | |
| ESOB2 | | 8.66 ± 1.76 | 5.98 ± 1.46 | 13.93 ± 2.75 | | 0.90 ± 0.16 | 0.60 ± 0.04 | 1.50 ± 0.20 | |

**Fig. S2** In vitro growth of wheat seedlings in presence of single PGPR


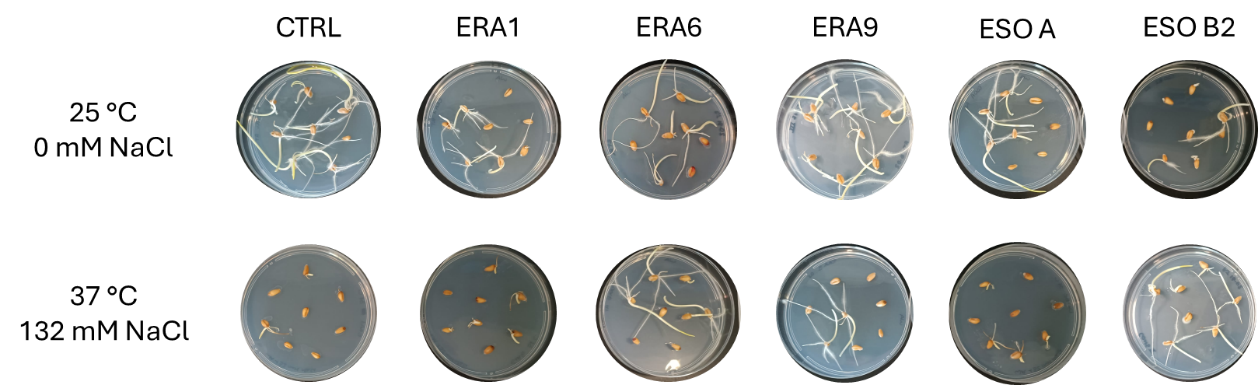


**Table S5** In vitro growth of wheat seedlings in presence of PGPR consortia

|  | **25 °C – 0 mM NaCl** | | | **37 °C – 132 mM NaCl** | | |
| --- | --- | --- | --- | --- | --- | --- |
| **Inoculation** | **Root length (cm)** | **Shoot length (cm)** | **Total length (cm)** | **Root length (cm)** | **Shoot length (cm)** | **Total length (cm)** |
| CTRL | 1.63 ± 0.44 | 1.28 ± 0.39 | 2.91 ± 0.71 | 0.43 ± 0.07 | 0.00 ± 0.00 | 0.43 ± 0.00 |
| CONSI | 1.94 ± 0.45 | 1.88 ± 0.31 | 3.81 ± 0.69 | 0.69 ± 0.06 | 0.00 ± 0.00 | 0.69 ± 0.06 |
| CONSII | 3.20 ± 0.58 | 2.41 ± 0.34 | 5.61 ± 0.89 | 0.71 ± 0.06 | 1.25 ± 0.20 | 1.27 ± 0.26 |
| CONSIII | 4.12 ± 0.50 | 3.23 ± 0.29 | 7.35 ± 0.69 | 0.60 ± 0.11 | 1.68 ± 0.46 | 2.21 ± 0.50 |

**Fig. S3** In vitro growth of wheat seedlings in presence of PGPR consortia

**
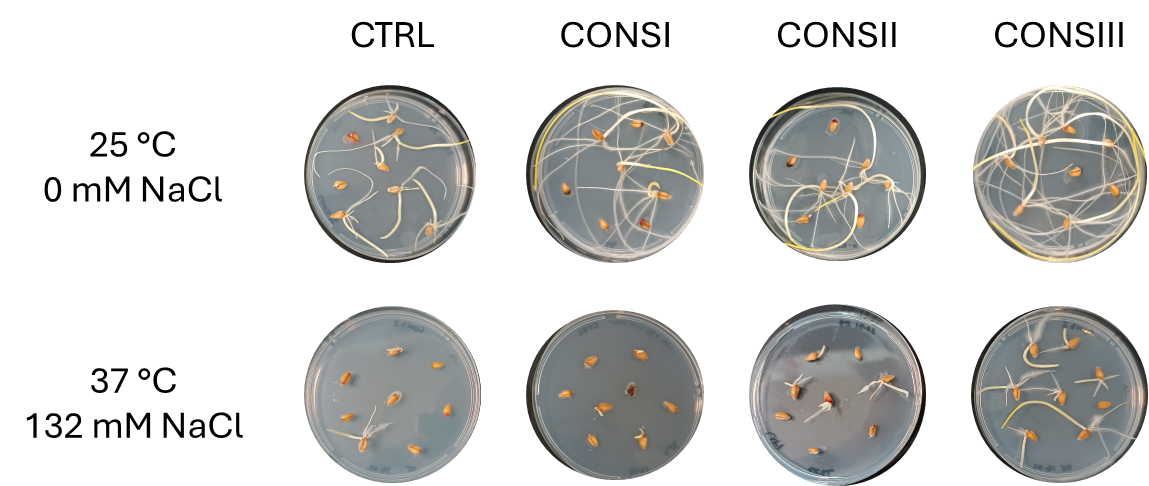
**

**Table S6** Plant growth-promoting activities of the strain composing CONSIII under three salt concentrations and three temperatures. Values of PVK, Protease activity, Cellulase activity and Chitinase activity are reported as d = halo diameter – colony diameter (d < 5 mm = +; d ≥ 5 mm = ++; d ≥ 10 mm = +++; d ≥ 15 mm = ++++; d ≥ 20 mm = +++++)

|  |  | **ERA6** | | | **ERA9** | | | **ESOB2** | | |
| --- | --- | --- | --- | --- | --- | --- | --- | --- | --- | --- |
|  | **[NaCl]** | **25 °C** | **37 °C** | **42 °C** | **25 °C** | **37 °C** | **42 °C** | **25 °C** | **37 °C** | **42 °C** |
| **PVK** | **50 mM** | **-** | **-** | **+** | **+** | **++** | **+** | **-** | **-** | **-** |
|  | **132 mM** | **-** | **-** | **-** | **++** | **++** | **+** | **-** | **-** | **-** |
|  | **330 mM** | **-** | **-** | **++** | **++** | **++** | **++** | **-** | **-** | **-** |
| **DPPH scavenging (%)** | **50 mM** | **62** | **62** | **39** | **58** | **59** | **37** | **79** | **65** | **38** |
|  | **132 mM** | **72** | **66** | **36** | **57** | **60** | **20** | **63** | **73** | **33** |
|  | **330 mM** | **72** | **45** | **39** | **58** | **35** | **36** | **63** | **42** | **36** |
| **NH_4_ production (µg/mL)** | **50 mM** | **488** | **324** | **97** | **218** | **186** | **109** | **307** | **302** | **221** |
|  | **132 mM** | **549** | **244** | **271** | **384** | **204** | **122** | **233** | **464** | **168** |
|  | **330 mM** | **137** | **308** | **170** | **93** | **94** | **116** | **221** | **332** | **181** |
| **IAA production (µg/mL)** | **50 mM** | **0** | **3** | **23** | **44** | **60** | **35** | **9** | **16** | **25** |
|  | **132 mM** | **2** | **4** | **5** | **49** | **87** | **39** | **17** | **20** | **2** |
|  | **330 mM** | **0** | **0** | **6** | **106** | **131** | **87** | **33** | **35** | **39** |
| **Protease activity** | **50 mM** | **++++** | **+++** | **-** | **-** | **-** | **-** | **++** | **++** | **-** |
|  | **132 mM** | **++++** | **+++** | **+** | **-** | **-** | **-** | **+++** | **++** | **-** |
|  | **330 mM** | **+++** | **++** | **-** | **-** | **-** | **-** | **+++** | **++** | **-** |
| **Cellulase activity** | **50 mM** | **-** | **-** | **-** | **-** | **-** | **-** | **++** | **++** | **++** |
|  | **132 mM** | **-** | **-** | **-** | **-** | **-** | **-** | **+++** | **+++** | **++++** |
|  | **330 mM** | **-** | **-** | **-** | **-** | **-** | **-** | **+++** | **++++** | **-** |
| **Amylase activity** | **50 mM** | **-** | **-** | **-** | **-** | **-** | **-** | **+++** | **++++** | **+++** |
|  | **132 mM** | **+** |  |  | **-** | **-** | **-** | **++++** | **+++** | **+++** |
|  | **330 mM** | **-** | **-** | **-** | **-** | **-** | **-** | **++** | **++++** | **++++** |
| **Chitinase activity** | **50 mM** | **+** | **+** | **+** | **+** | **+** | **+** | **-** | **-** | **-** |
|  | **132 mM** | **+** | **+** | **+** | **+** | **+** | **+** | **-** | **-** | **-** |
|  | **330 mM** | **+** | **+** | **-** | **+** | **+** | **+** | **-** | **-** | **-** |
